# Alendronic Acid as Ionic Liquid: New Perspective on Osteosarcoma

**DOI:** 10.3390/pharmaceutics12030293

**Published:** 2020-03-24

**Authors:** Sónia Teixeira, Miguel M. Santos, Maria H. Fernandes, João Costa-Rodrigues, Luís C. Branco

**Affiliations:** 1 Faculdade de Medicina Dentária, U. Porto, Rua Dr. Manuel Pereira da Silva, 4200-393 Porto, Portugal; soniateixeira.88@gmail.com (S.T.); mhfernandes@fmd.up.pt (M.H.F.); joao.m.rodrigues@gmail.com (J.C.-R.); 2 LAQV-REQUIMTE, Departamento de Química, Faculdade de Ciências e Tecnologia, Universidade Nova de Lisboa, 2829-516 Caparica, Portugal; miguelmsantos@fct.unl.pt; 3 UCIBIO-REQUIMTE, Departamento de Química, Faculdade de Ciências, Universidade do Porto, 4169-007 Porto, Portugal; 4 ESS—Escola Superior de Saúde, Instituto Politécnico do Porto, R. Dr. António Bernardino de Almeida 400, 4200-072 Porto, Portugal; 5 Instituto Politécnico de Viana do Castelo, Escola Superior de Saúde, Rua D. Moisés Alves Pinho 190, 4900-314 Viana do Castelo, Portugal

**Keywords:** alendronic acid, Active Pharmaceutical Ingredients, API-OSILs, anticancer drugs, ionic liquids

## Abstract

Herein the quantitative synthesis of eight new mono- and dianionic Organic Salts and Ionic Liquids (OSILs) from alendronic acid (ALN) is reported by following two distinct sustainable and straightforward methodologies, according to the type of cation. The prepared ALN-OSILs were characterized by spectroscopic techniques and their solubility in water and biological fluids was determined. An evaluation of the toxicity towards human healthy cells and also human breast, lung and bone (osteosarcoma) cell lines was performed. Globally, it was observed that the monoanionic OSILs showed lower toxicity than the corresponding dianionic structures to all cell types. The highest cytotoxic effect was observed in OSILs containing a [C_2_OHMIM] cation, in particular [C_2_OHMIM][ALN]. The latter showed an improvement in IC_50_ values of ca. three orders of magnitude for the lung and bone cancer cell lines as well as fibroblasts in comparison with ALN. The development of OSILs with high cytotoxicity effect towards the tested cancer cell types, and containing an anti-resorbing molecule such as ALN may represent a promising strategy for the development of new pharmacological tools to be used in those pathological conditions.

## 1. Introduction

Alendronic acid is an aminobisphosphonate derivative that has shown efficacy in postmenopausal osteoporosis, malignant hypercalcemia and Paget’s disease [1]. Alendronate localizes preferentially at active sites of bone resorption, which has been inhibited at doses that have no effect on bone mineralization [2].

Bisphosphonates bind at the bone mineral surface, where they potently inhibit osteoclast-mediated bone resorption and subsequently embed in the bone, being released only during subsequent resorption [3]. In contrast to other antiresorptive agents, bisphosphonates with the greatest binding affinity to bone (zoledronic acid > alendronate > ibandronate > risedronate) may persist in bone, and patients continue to be exposed to the pharmacologic effects of these drugs several years after discontinuation.

All bisphosphonates rapidly reduce bone resorption, which leads to decreased bone formation because resorption and formation are coupled. Within three to six months, equilibrium is reached at a lower rate of bone turnover [4].

It is described that alendronate in rats exhibited 200 to 1000 times more potency than etidronate and approximately 100 times more in comparison with clodronate or tiludronate [5]. It is recognized that the presence of amino group side-chain from alendronate chemical structure contributes to greater potency and specificity [3,4,5].

The introduction of specific functional groups in the BPs structure can lead to modifications in their physicochemical, biological, therapeutic and toxicological properties. In recent literature studies, alendronic acid is reported as an FDA drug already approved for the prevention and treatment of osteoporosis in men and women, either postmenopausal or glucocorticoid-induced [6]. However, BPs present low bioavailability when administered orally and frequently require parenteral administration, which is not the most convenient route in case of continuous treatment.

The combination of Active Pharmaceutical Ingredients (APIs) with biocompatible organic counter-ions has rendered over the last decade an attractive class of materials entitled API–Organic Salts and Ionic Liquids (API-OSILs) [7,8,9,10]. Ionic Liquids (ILs) are defined as salts with a melting point below 100 °C, which display peculiar properties such as negligible vapor pressure, high thermal and chemical stability and tunable physicochemical properties according to the cation-anion combinations. These novel API–OSILs can improve the drug performance in terms of stability, solubility, permeability, biological activity and delivery [10,11,12,13,14,15,16,17,18,19,20,21,22]. Recent achievements showed that the suitable combination between different families of pharmaceutical drugs such as NSAIDs (e.g., ibuprofen and naproxen [15,16]), β-lactam (e.g., ampicillin, amoxicillin, penicillin [16,17,18,19,20]) and fluoroquinolone (ciprofloxacin, norfloxacin [16,21]) antibiotics and bone antiresorptive agents (zoledronic acid [22]) rendered novel pharmaceutical drug formulations based on OSILs. Elimination of original polymorphism, improvement of permeability and solubility in water and biological fluids as well as increased therapeutic efficiency was observed for these innovative compounds.

Thus, considering the pharmaceutical importance of bisphosphonate drugs as antiresorptive and potential antitumoral agents, it is of paramount importance that novel formulations of such drugs, which render higher bioavailability and lower systemic toxicity than the latter are developed. Hence, in this paper, we report our latest results on our research line regarding bisphosphonate-based OSILs. In particular, we describe the synthesis of eight new Ionic Liquids and Organic Salts from alendronic acid (ALN–OSILs) as mono- and dianion by combination with one or two units of biocompatible cations, respectively. The desired compounds were prepared in quantitative yields by two distinct sustainable and straightforward methodologies, according to the type of cation. All prepared ALN–OSILs were characterized by spectroscopic techniques and their solubility in water and biological fluids was determined. Finally, evaluation of the toxicity towards human healthy cells and lung, breast and bone (osteosarcoma) cell lines was performed.

## 2. Synthesis and Characterization of ALN–OSILs

**Figure 1:**
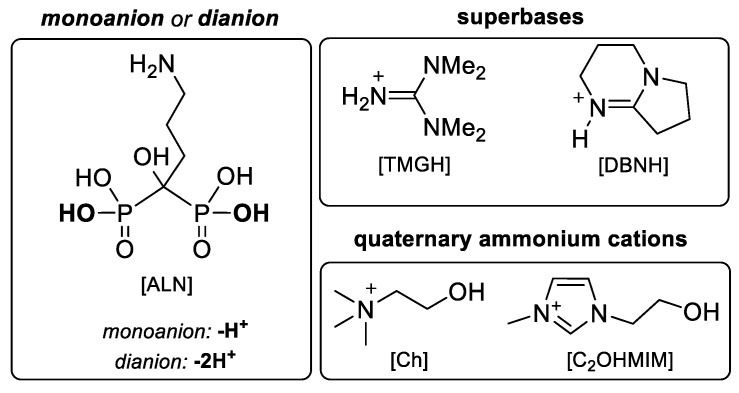
Structure of mono- and dianionic alendronate, protonated superbases and organic quaternary cations used to prepare the alendronic acid (ALN)–Organic Salts and Ionic Liquids (OSILs).

Figure 1 depicts the structures of ALN and the selected cations, which were combined in 1:1 or 1:2 stoichiometric ratios, thereby deprotonating one or two phosphonate groups, respectively.

The selection of protonated organic superbases (1,1,3,3-tetramethylguanidinium [TMGH] and 1,5-diazabicyclo(4.3.0)non-5-enium [DBNH]) and quaternary ammonium (cholinium [Ch] and 1-(2-hydroxyethyl)-3-methyl-1*H*-imidazol-3-ium [C_2_OHMIM]) cations relied on the known information about their biocompatibility [15,23]. Despite their low toxicity, the combination of these cations with other APIs has rendered highly active API–OSILs in the past, in particular antimicrobial and anti-tumoral [19,20,22].

**Scheme 1:**
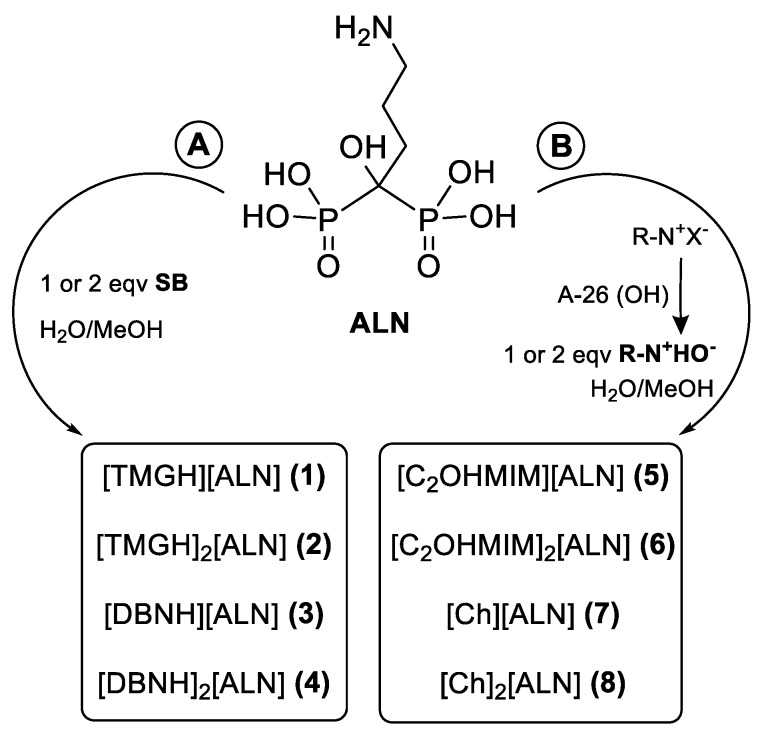
Methodologies (**A**,**B**) for the preparation of ALN–OSILs.

Scheme 1 resumes the synthetic methodologies employed in the synthesis of the API–OSILs.

The preparation of compounds **1**–**4** consisted of the addition of the diluted superbases to a dispersion of ALN (pathway A). These particular compounds, termed Superionic Liquids of APIs, were prepared according to a previous methodology reported by us for zoledronic acid [22] as well as other APIs [16]. While both monoanionic ALN–OSILs were obtained as solids, the two dianionic were isolated as pastes.

On the other hand, a combination of ALN with one and two equivalents of [Ch] and [C_2_OHMIM] cations yielded four more ALN-based OSILs (compounds **5**–**8**) by following another methodology already described by our group [15,22]. In this case, quaternary ammonium hydroxide cations are previously prepared by the corresponding halide exchange with hydroxide exchange resins (e.g., Amberlyst A26-OH) in methanolic solution. The very basic solutions were then neutralized by addition to an aqueous solution of bisphosphonate yielding the desired salts in quantitative yields. From the four synthesized compounds, two are RTILs while the other two are solids (see below).

All products were characterized by NMR (^1^H and ^13^C) and FTIR spectroscopic techniques, as well as elemental analysis. The thermal properties were evaluated by DSC and the solubility in water and saline solution was determined for all compounds.

**Figure 2:**
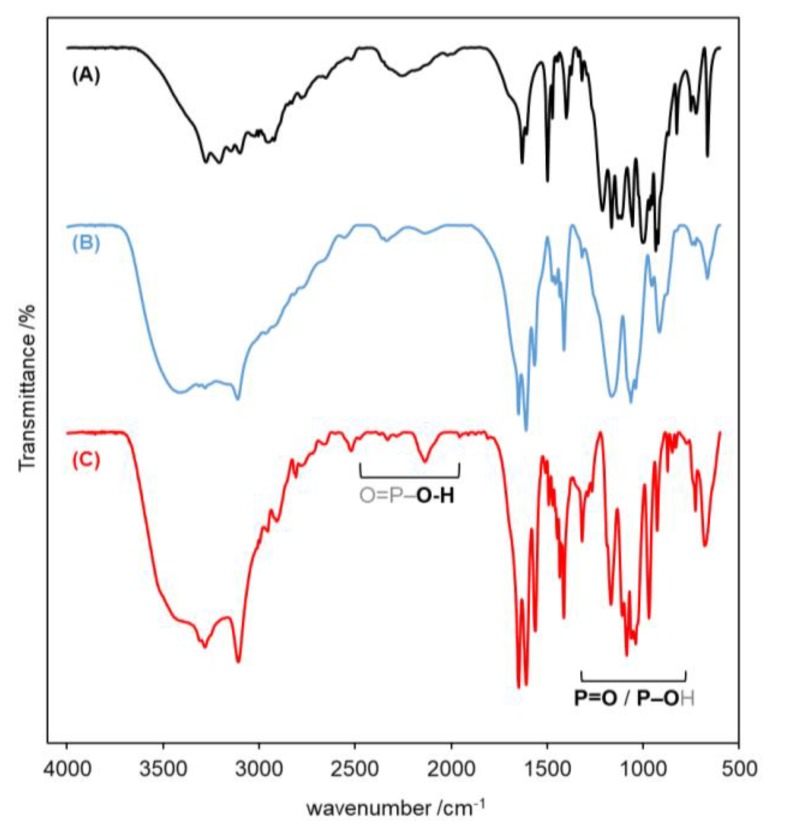
FTIR spectra of (**A**) ALN, (**B**) [TMGH][ALN] and (**C**) [TMGH]_2_[ALN].

The NMR spectra of the ALN–OSILs were acquired in D_2_O taking advantage of their high solubility in water (see below). In all cases, the ^1^H NMR spectra showed that the cation/anion proportion is strictly 1.0:1.0 or 2.0:1.0, in agreement with the intended stoichiometry (see Figures S1–S16). In addition, only one set of signals was observed, meaning that the reactions were complete and only one product was formed. No comparison with alendronic acid is achievable because of its lack of solubility in the same solvents as the ALN–OSILs. In the ^13^C NMR spectra, the resonance of the quaternary carbon atom of ALN appears at ca. 74 ppm, with no particular difference if only one or two neighboring phosphonate groups are deprotonated. Similarly, the ^1^H NMR data is also irreflective of the ionic state of the bisphosphonates and also of the cations. In contrast, the collected FTIR spectra (Figures S17–S25) show pristine variations between the neutral bisphosphonates and the ALN–OSILs, as well as between mono- and dianions. The FTIR spectrum of ALN shows two characteristic regions, namely a weak and undefined structure at 2400–2000 cm^−1^ with maximum intensity at 2256 cm^−1^, and also very intense multiple peaks at 1250–900 cm^−1^ (see Figure 2A). While the first one accounts for O–H stretches from the O=P–OH groups, the second region contains peaks assignable to the stretch of both P=O and P–OH bonds [24]. In the spectra of the synthesized ALN–OSILs (see Figure 2 for [TMGH]-based ALN–OSILs), the peaks in the first region become sharper and much weaker, and two other sets of peaks appear in the vicinity (at ca. 2400–2300 cm^−1^ and 2200–2100 cm^−1^) which are more intense for the dianionic ALN–OSILs than for the monoanionic. This is in complete agreement with changes in the vibrational modes of the OH groups from the phosphonate moieties that are particularly enhanced when both groups are deprotonated. This corollary is also sustained by the changes observed in the second region of peaks, which in general displays two very intense broad peaks at ca. 1160 cm^−1^ and 1060 cm^−1^, and one or two of medium intensity between 960 and 900 cm^−1^.

## 3. Thermal Analysis of ALN–OSILs

All prepared OSILs from alendronic acid were studied by Differential Scanning Calorimetry (DSC) techniques (see Figures S26–S33). Table 1 contains the obtained data, namely melting, crystallization and glass transition temperatures, as well as the physical state of the analyzed compounds.

In general, the monoanionic ALN–OSILs are foam-like solids while the dianionic ones were obtained as thick colorless pastes at room temperature, thus being considered Room Temperature Ionic Liquids (RTILs). The exceptions to this rule are the compounds with the [C_2_OHMIM] cation, where the mono- and dianionic are, respectively, a paste and a foam. This inversion of the trend is probably related to specific interactions of ALN with the electron-rich imidazolium ring [25].

**Figure 3:**
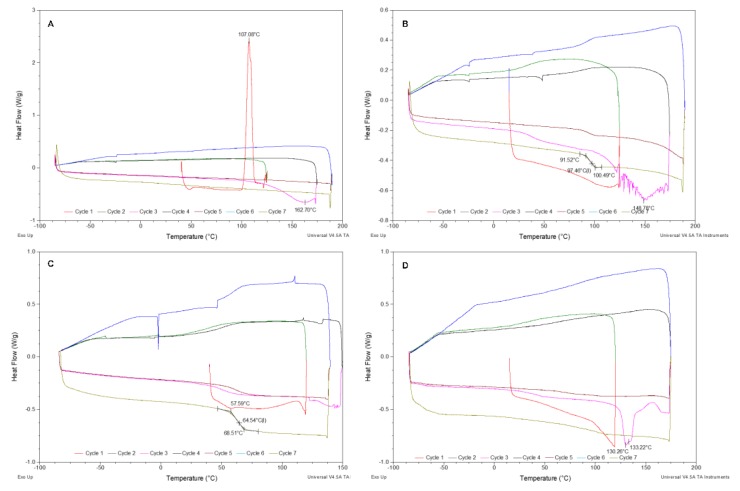
DSC thermograms of (**A**) [TMGH][ALN], (**B**) [TMGH]_2_[ALN], (**C**) [C_2_OHMIM][ALN] and (**D**) [DBNH][ALN].

In comparison with sodium alendronate, which presents a melting temperature of 259.3 °C [26], all solid compounds melt at lower temperatures, more specifically between 130.3 and 162.7 °C, for [DBNH]- and [TMGH][ALN] OSILs, respectively. These melting temperatures are determined in the third cycle of the experiment, which consists of heating from −90 °C to 150–190 °C (depending on the compound) at 10 °C/min. The first two cycles typically consist of heating to 125 °C and isotherm for 15–20 min (for the complete removal of residual water) followed by cooling to −90 °C. From Cycle 3 onwards, consecutive cooling/heating cycles are performed at 10 or 20 °C/min. These cycles show glass transition temperatures (T_g_) for the majority of the ALN–OSILs, showing that they become supercooled products, i.e., amorphous after the first melt. The exceptions to this behavior are [TMGH] and [DNBH][ALN], to which no glass transition nor crystallization (T_c_) temperatures were observed in the experimental conditions. However, [TMGH][ALN] showed different behavior in comparison with the remaining ALN–OSILs in the first cycle, in particular a cold crystallization (T_cc_) peak at 107.1 °C (Figure 3A).

The DSC thermogram of the RTIL [TMGH]_2_[ALN] (Figure 3B) showed an endothermic signal at ca. 150 °C of the third cycle that could be assigned to a melting process. However, it is preceded by a glass transition at ca. 40 °C in the same cycle, meaning that the compound is in an amorphous state. So, the referred endothermic signal is most likely due to evaporation, consistent with the irregular shape of the curve caused by the formation of bubbles in the thick pasty compound. A similar observation can also be observed in [C_2_OHMIM][ALN] (Figure 3C). From the set of eight compounds, [DBNH][ALN] is the only one that possesses a polymorphic structure, given by the two melting temperatures at 130.3 and 133.2 °C recorded in the DSC thermogram (Figure 3D). The remaining RTILs, more precisely [DBNH]_2_[ALN] and [Ch]_2_[ALN] show well-defined glass transitions confirming their amorphous nature at room temperature (Figures S29 and S33, respectively).

## 4. Solubility Studies

**Figure 4:**
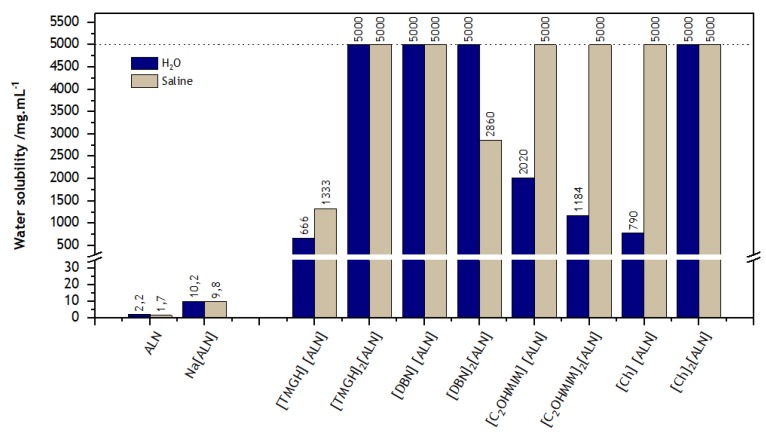
Solubility in water and saline solution at 37 °C of ALN, Na[ALN] and corresponding ALN–OSILs (detection limit is 5 g/mL, represented by the upper threshold).

As expected, all OSILs were more soluble in water and saline solution at 37 °C than alendronic acid as well as its sodium salt. Figure 4 contains the data obtained from the solubility studies.

With the exception of the [DBNH]- and [C_2_OHMIM]-containing ILs, the dianionic ALN–OSILs are equally or more soluble than the monoanionic siblings in the tested media. In further detail, it is noteworthy that [TMGH]_2_[ALN], [DBNH][ALN] and [Ch]_2_[ALN] are completely soluble in both water and saline solution in the tested conditions. Moreover, the [C_2_OHMIM]-, [C_2_OHMIM]_2_- and [Ch]-based ALN–OSILs are also fully soluble in saline solution while the solubility in water was found to be between 392 and 918 times higher in comparison with the parent neutral drug, and between 77 and 198 times when compared with Na[ALN]. In addition, [DBNH]_2_[ALN] presents full water solubility and a 1682-fold increase in solubility in saline solution. Finally, [TMGH][ALN] shows the lowest solubility in both media from the set of eight synthesized compounds.

## 5. Cytotoxicity on Human Cells

The cytotoxicity of the prepared ALN–OSILs was determined on human cells, by means of IC_50_ calculation. The analysis was performed on human gingival fibroblasts (GF) and on different cancer cell lines, namely T47D (breast), A549 (lung) and MG63 (osteosarcoma). The results obtained with the starting materials of the synthesis, as well as the prepared ALN–OSILs upon incubation for 24 h are presented in Table 2. The cytotoxic effect of the ALN-OSILs on the cancer cell lines was also evaluated after 72h of exposure, and the data is presented in Table 3. A comparison with the standard anticancer drug paclitaxel is presented for both exposure times.

Globally, it was observed that the IC_50_ values determined at 72 h of incubation were higher than those obtained with a culture period of 24 h. The IC_50_ values obtained for paclitaxel in the tumor cell lines were the lowest ones, which is in line with its well-known cytotoxic potential. However, it showed no selectivity between healthy and cancer cells.

In the case of the ALN–OSILs, the obtained data clearly shows that the monoanionic OSILs elicited lower cytotoxicity than the corresponding dianionic versions for all cell types. Regarding gingival fibroblast cell cultures, the OSILs containing one unit of [DBNH], [C_2_OHMIM] and [Ch] appeared to be less deleterious than alendronic acid. Comparatively, OSILs containing [TMGH]_2_, [C_2_OHMIM] and [C_2_OHMIM]_2_ seemed to be more cytotoxic to breast cancer T47D cells. In the case of lung cancer A549 cell line, high cytotoxic activity was observed in cell cultures supplemented with the OSILs containing [TMGH]_2_, [C_2_OHMIM] and [Ch]. Finally, osteosarcoma MG63 cell line seemed to be particularly sensitive to the OSILs containing [TMGH]_2_, [C_2_OHMIM] and [C_2_OHMIM]_2_.

Breast and lung cancers are often associated with bone osteolytic metastases [27,28,29] and also osteosarcoma, which are usually caused by disturbances in bone metabolism and increases in bone turnover rates [6,30,31]. Thus, the development of OSILs with high cytotoxicity towards the tested cancer cell types and containing an anti-resorbing molecule (alendronate) may represent a promising strategy for the development of new pharmacological tools to be used in those pathological conditions.

Overall, [C_2_OHMIM][ALN] was found to be particularly active against lung cancer and osteosarcoma cell lines while retaining very low toxicity towards healthy cells. These enhanced biological properties, in addition to the absence of polymorphism in this monoanionic compound, suggest that this room temperature ionic liquid could be a very promising alendronic acid formulation.

## 6. Experimental Section

### 6.1. General Procedure (A) for the Synthesis of ALN–OSILs with Organic Superbases as Cations

To a dispersion of alendronic acid (400 mg, 1.61 mmol) in MeOH/H_2_O (15 mL, 1:1) a methanolic solution of 1 or 2 molar equivalents of organic superbase (15 mg/mL) was added dropwise at room temperature under magnetic stirring. After reacting for 1 h, the solvent was evaporated and the desired product was dried under vacuo for 24 h.

### 6.2. General Procedure (B) for the Preparation of ALN–OSILs with Ammonium and Methylmidazolium Cations

The halide salts of the selected ammonium and methylimidazolium cations were dissolved in methanol and passed slowly through an anion-exchange column A-26(OH) (3 equivalents). The freshly formed methanolic solutions of the corresponding hydroxide salts (1 or 2 equivalents) were consequently added dropwise to alendronic acid (400 mg, 1.61 mmol) dispersed in H_2_O under magnetic stirring at room temperature. After 1 h, the solvent of the clear solution was evaporated, and the desired product was dried under vacuo for 24 h.

Toxicity studies are described as supporting information.

## Figures and Tables

**Table 1:** Physical state, melting (T_m_), cold crystallization (T_cc_) and glass transition (T_g_) temperatures of the prepared ALN–OSILs.

Salt	Physical State	T_m_/°C	T_cc_/°C *	T_g_/°C
[TMGH][ALN]	White solid	162.7	107.1	-
[TMGH]_2_[ALN]	Colorless paste	-	-	97.5
[DBNH][ALN]	White solid	130.3; 133.2	-	-
[DBNH]_2_[ALN]	Colorless paste	-	-	45.7
[C_2_OHMIM][ALN]	Colorless paste	-	-	64.5
[C_2_OHMIM]_2_[ALN]	White solid	153.0 (dec)	-	46.3
[Ch][ALN]	White solid	141.2	-	74.9
[Ch]_2_[ALN]	Colorless paste	-	-	63.8

* Cold crystallization.

**Table 2:** Cytotoxicity of the ALN–OSILs on GF cells and T47D, A549 and MG63 cell lines after 24 h.

	IC_50_/mM			
IL	GF	T47D	A549	MG63
Paclitaxel	1.91 × 10^−5^ ± 0.34 × 10^−5^	6.46 × 10^−6^ ± 0.57 × 10^−6^	4.08 × 10^−6^ ± 0.60 × 10^−6^	8.19 × 10^−6^ ± 1.03 × 10^−6^
ALN	3.17 × 10^−2^ ± 0.12 × 10^−2^	4.09 × 10^−3^ ± 0.12 × 10^−3^ (*)	8.10 × 10^−3^ ± 0.88 × 10^−3^ (*)	5.55 × 10^−4^ ± 0.79 × 10^−4^ (*)
[TMGH]Cl	1.47 × 10^−3^ ± 0.40 × 10^−3^	1.94 × 10^−4^ ± 0.35 × 10^−4^	4.98 × 10^−4^ ± 0.55 × 10^−4^	2.46 × 10^−4^ ± 0.81 × 10^−4^
[DBNH]Cl	3.80 × 10^−5^ ± 0.53 × 10^−5^	2.78 × 10^−4^ ± 0.19 × 10^−4^	9.13 × 10^−3^ ± 1.03 × 10^−3^	9.79 × 10^−8^ ± 1.76 × 10^−8^
[Ch]Cl	(a)	(a)	5.99 × 10^−3^ ± 0.72 × 10^−3^	3.47 × 10^−4^ ± 0.52 × 10^−4^
[TMGH][ALN]	7.19 × 10^−3^ ± 0.97 × 10^−3^	8.23 × 10^−3^ ± 0.99 × 10^−3^	2.54 × 10^−3^ ± 0.32 × 10^−3^	9.23 × 10^−3^ ± 1.12 × 10^−3^
[TMGH]_2_[ALN]	1.11 × 10^−3^ ± 0.22 × 10^−3^	3.16 × 10^−5^ ± 0.25 × 10^−5^	5.14 × 10^−4^ ± 0.71 × 10^−4^	7.11 × 10^−5^ ± 0.85 × 10^−5^
[DBNH][ALN]	7.20 × 10^−2^ ± 0.55 × 10^−2^	(a)	6.14 ± 0.87	(a)
[DBNH]_2_[ALN]	6.01 × 10^−5^ ± 0.94 × 10^−5^	(a)	3.12 × 10^−2^ ± 0.45 × 10^−2^	1.02 ± 0.18
[C_2_OHMIM][ALN]	2.19 ± 0.32	5.92 × 10^−3^ ± 0.69 × 10^−3^ (*)	2.10 × 10^−6^ ± 0.37 × 10^−6^ (*)	5.16 × 10^−5^ ± 0.67 × 10^−5^ (*)
[C_2_OHMIM]_2_[ALN]	4.07 × 10^−4^ ± 0.47 × 10^−4^	3.28 × 10^−6^ ± 0.40 × 10^−6^ (*)	(a)	7.84 × 10^−5^ ± 0.98 × 10^−5^ (*)
[Ch][ALN]	1.87 × 10^−1^ ± 0.32 × 10^−1^	3.14 ± 0.43	6.66 × 10^−2^ ± 0.89 × 10^−2^ (*)	4.10 × 10^−1^ ± 0.62 × 10^−1^
[Ch]_2_[ALN]	4.92 × 10^−3^ ± 0.58 × 10^−3^	2.64 × 10^−3^ ± 0.38 × 10^−3^	5.41 × 10^−3^ ± 0.61 × 10^−3^	1.01 × 10^−2^ ± 0.14 × 10^−2^

(a) Not determined in the tested concentration range. (*) Significantly lower than GF; *p* < 0.05.

**Table 3:** Cytotoxicity of the ALN–OSILs on GF cells and T47D, A549 and MG63 cell lines after 72 h.

	IC_50_/mM			
IL	GF	T47D	A549	MG63
Paclitaxel	5.66 × 10^−5^ ± 0.94 × 10^−5^	9.28 × 10^−6^ ± 1.02 × 10^−6^	7.83 × 10^−6^ ± 0.85 × 10^−6^	1.10 × 10^−6^ ± 1.25 × 10^−6^
ALN	9.26 × 10^−2^ ± 0.99 × 10^−2^	8.93 × 10^−3^ ± 1.53 × 10^−3^ (*)	1.46 × 10^−2^ ± 0.43 × 10^−2^ (*)	1.07 × 10^−3^ ± 0.26 × 10^−3^ (*)
[TMGH][ALN]	1.33 × 10^−2^ ± 0.51 × 10^−2^	1.84 × 10^−2^ ± 0.24 × 10^−2^	6.16 × 10^−3^ ± 0.94 × 10^−3^	1.64 × 10^−2^ ± 0.82 × 10^−2^
[TMGH]_2_[ALN]	1.68 × 10^−3^ ± 0.27 × 10^−3^	1.87 × 10^−5^ ± 0.43 × 10^−5^ (*)	9.84 × 10^−4^ ± 0.67 × 10^−4^	1.67 × 10^−4^ ± 0.25 × 10^−4^
[DBNH][ALN]	2.55 × 10^−1^ ± 0.34 × 10^−1^	(a)	0.99 ± 0.07	(a)
[DBNH]_2_[ALN]	1.65 × 10^−4^ ± 0.25 × 10^−5^	(a)	9.33 × 10^−2^ ± 1.00 × 10^−2^	1.14 ± 0.21
[C_2_OHMIM][ALN]	4.54 ± 0.66	1.14 × 10^−2^ ± 0.25 × 10^−2^ (*)	5.65 × 10^−6^ ± 0.46 × 10^−6^ (*)	9.57 × 10^−5^ ± 0.91 × 10^−5^ (*)
[C_2_OHMIM]_2_[ALN]	6.21 × 10^−4^ ± 0.67 × 10^−4^	8.83 × 10^−6^ ± 0.94 × 10^−6^ (*)	(a)	1.65 × 10^−4^ ± 0.26 × 10^−4^ (*)
[Ch][ALN]	7.33 × 10^−1^ ± 0.63 × 10^−1^	4.86 ± 0.55	7.84 × 10^−2^ ± 0.83 × 10^−2^ (*)	6.85 × 10^−1^ ± 0.85 × 10^−1^
[Ch]_2_[ALN]	8.57 × 10^−3^ ± 0.87 × 10^−3^	4.87 × 10^−3^ ± 0.65 × 10^−3^	8.94 × 10^−3^ ± 1.34 × 10^−3^	8.33 × 10^−2^ ± 0.93 × 10^−2^

(a) Not determined in the tested concentration range. (*) Significantly lower than GF, *p* < 0.05.

## Supplementary Materials

The following are available online at https://www.mdpi.com/1999-4923/12/3/293/s1, Materials, experimental procedures, compound characterization data. Figures S1–S16: NMR spectra of ALN-OSILs; Figures S17–S25: FTIR spectra of ALN and ALN-OSILs; Figures S26–S33: DSC thermograms of ALN-OSILs.

## Abbreviations

IL	ionic liquid
ALN	alendronic acid
ALN–OSILs	alendronic acid-based ionic liquids and organic salts
RTILs	Room Temperature Ionic Liquids
API–OSILs	Active Pharmaceutical Ingredient Ionic Liquids and Organic Salts
TMG	1,1,3,3-tetramethylguanidine
DBN	1,5-diazabicyclo(4.3.0)non-5-ene
Ch	choline
C_2_OHMIM	1-(2-hydroxyethyl)-3-methylimidazolium
