# Supplementary material for: Alendronic Acid as Ionic Liquid: New Perspective on Osteosarcoma
Source: Pharmaceutics. 2020 Mar 24;12(3):293. doi: 10.3390/pharmaceutics12030293 (PMC7151258; doi:10.3390/pharmaceutics12030293)
Supplement: Supplementary file 1 [file pharmaceutics-12-00293-s001.pdf]

pharmaceutics

Supplementary Materials: Alendronic Acid as Ionic Liquid: New Perspective on Osteosarcoma

Sónia Teixeira, Miguel M. Santos, Maria H. Fernandes, João Costa-Rodrigues and Luís C. Branco

Experimental

Materials

All acquired reagents were used without further purification. Alendronic acid (ALN, ≥98.5%) was purchased from Molekula, 1,1,3,3-tetramethylguanidine (TMG, 99%), 1,5-diazabicyclo(4.3.0)non-5-ene (DBN, 99%), and choline chloride (ChCl, 99%), were supplied by Sigma-Aldrich, and 1-(2-hydroxyethyl)-3-methylimidazolium chloride ([C_2OHMIM]Cl], 98%), was purchased at Solchemar. Methanol HPLC grade was acquired from Honeywell and deionized water was processed by Diwer Technologies water max w2 equipment.

General Procedure (A) for the Synthesis of ALN-OSILs with Organic Superbases as Cations:

To a dispersion of alendronic acid (400 mg, 1.61 mmol) in MeOH/H_2O (15 mL, 1:1) a methanolic solution of 1 or 2 molar equivalents of organic superbase (15 mg/mL) was added dropwise under magnetic stirring. After reacting for 1h the solvent was evaporated and the desired product was dried under vacuo for 24 h.

General Procedure (B) for the Preparation of ALN-OSILs with Ammonium and Methylimidazolium Cations:

The halide salts of the selected ammonium and methylimidazolium cations were dissolved in methanol and passed slowly through an anion-exchange column A-26(OH) (3 equivalents). The freshly formed methanolic solutions of the corresponding hydroxide salts (1 or 2 equivalents) were consequently added dropwise to alendronic acid (400 mg, 1.61 mmol) dispersed in H_2O under magnetic stirring. After 1 h, the solvent of the clear solution was evaporated and the desired product was dried under vacuo for 24 h.

Characterization

The prepared compounds were characterized by ^{1}H and ^{13}C NMR recorded on a Bruker AMX400 spectrometer. Chemical shifts are reported downfield in parts per million considering the solvent residual signal. ^{13}C NMR spectra in D_2O were referenced to added MeOH or MeCN. IR spectra were recorded on a FTIR Bruker Tensor 27 Spectrometer using KBr matrixes. DSC analysis was carried out using a TA Instruments Q-series TM Q2000 DSC with a refrigerated cooling system. Between 2 and 10 mg of salt were crimped into an aluminum standard sample pan with lid which was continuously purged with nitrogen gas at 50 mL/min. The employed procedure was dependent on the melting point of the sample. A typical experiment consisted on a heating step (20 °C/min) to 125 °C (15–20 minutes), cooling (20 °C/min) to −90 °C, heating (10 °C/min) to 200 °C, cooling (10 °C/min) to −90 °C, heating (10 °C/min) to 200 °C, cooling (10 °C/min) to –90 °C, heating (20 °C/min) to 200 °C and cooling (20 °C/min) to −90 °C. Glass transition (T_g), melting (T_m) cold crystallization (T_{cc}) and decomposition temperatures were determined in the heating steps, while crystallization temperatures (T_c) were acquired in the cooling steps. The solubility of the salts in water and saline solution was determined by adding 5 to 10 μL of solvent to an Eppendorf containing precisely weighed ca. 30 mg of sample until a homogeneous solution is obtained upon mixture in a vortex.

Experimental Data of the Synthesized Compounds

Preparation of bis(dimethylamino)methaniminium hydrogen (4-amino-1-hydroxy-1-phosphonobutyl)phosphonate, [TMGH][ALN] (**1**)

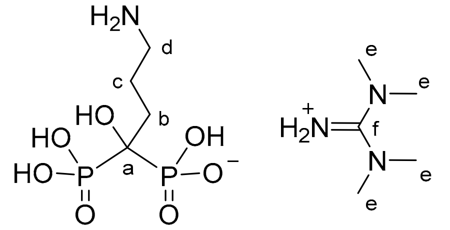

Using tetramethylguanidine (185 mg, 1.61 mmol) [TMGH][ALN] was obtained as a white solid in quantitative yield (585 mg). T_m = 48.1, 162.7 °C, T_{cc} = 107.1 °C; ^{1}H NMR (400.13 MHz, D_2O) δ 3.07–2.99 (m, 2H, d), 2.93 (s, 12H, e), 2.06–1.92 (m, 4H, b, c). ^{13}C NMR (100.62 MHz, D_2O) δ 162.0 (f), 74.1 (t, J = 135.3 Hz, a), 40.6 (d), 39.5 (e), 31.2 (c), 22.8 (t, J = 6.8 Hz, b) ppm; FTIR (KBr) 3407, 3112, 2966, 2818, 2337, 2137, 1649, 1610, 1566, 1412, 1164, 1064, 1039, 955, 916 cm^{-1}. Anal. calcd for $C_9H_{26}N_4O_7P_2.2H_2O$: C, 27.00; H, 7.55; N, 14.00; found: C, 27.60; H, 8.02; N, 14.25.

Preparation of bis(bis(dimethylamino)methaniminium) (4-amino-1-hydroxybutane-1,1-diyl)bis(hydrogen phosphonate), [TMGH]$_2$[ALN] (**2**)

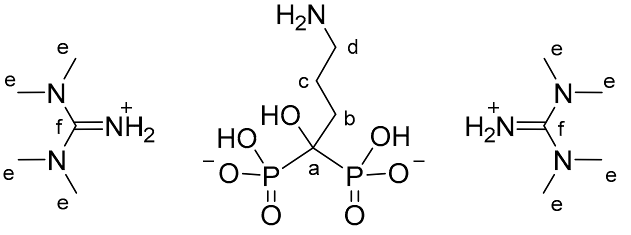

Using tetramethylguanidine (370 mg, 3.22 mmol) [TMGH]$_2$[ALN] was obtained as a colorless paste in quantitative yield (770 mg). T_m = 148.8 °C, T_g = 97.5 °C; ^{1}H NMR (400.13 MHz, D_2O) δ 3.07–2.98 (m, 2H, d), 2.93 (s, 24H, e), 2.02–1.88 (m, 4H, b, c); ^{13}C NMR (100.62 MHz, DMSO-*d*^{6}) δ 162.1 (f), 74.2 (t, J = 135.1 Hz, a), 40.7 (d), 39.5 (e), 31.7 (c), 23.1 (t, J = 7.1 Hz, b) ppm; FTIR (KBr) 3282, 3109, 2955, 2908, 2808, 2658, 2520, 2330, 2136, 1956, 1648, 1609, 1561, 1413, 1318, 1168, 1086, 1039, 970, 927 cm^{-1}. Anal. calcd for $C_{14}H51N_7O_{13}P_2.6H_2O$: C, 28.62; H, 8.75; N, 16.69; found: C, 28.58; H, 8.86; N, 16.69.

Preparation of 2,3,4,6,7,8-hexahydropyrrolo[1,2-a]pyrimidin-1-ium hydrogen (4-amino-1-hydroxy-1-phosphonobutyl)phosphonate, [DBNH][ALN] (**3**)

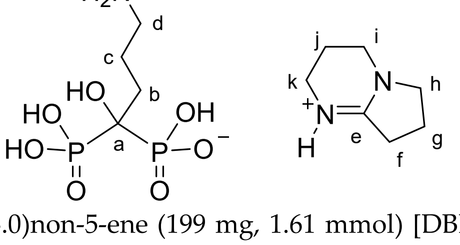

Using 1,5-diazabicyclo(4.3.0)non-5-ene (199 mg, 1.61 mmol) [DBNH][ALN] was obtained as a white solid in quantitative yield (598 mg). T_m = 130.3, 133.2 °C; ^{1}H NMR (400.13 MHz, D_2O) δ 3.64 (t, J = 7.3 Hz, 2H, k), 3.39 (t, J = 5.6 Hz, 2H, h), 3.34 (t, J = 5.6 Hz, 2H, i), 3.07–2.98 (m, 2H, d), 2.82 (t, J = 8.0 Hz, 2H, f), 2.10 (quint, J = 7.6 Hz, 2H, g), 2.04–1.92 (m, 6H, b, c, j). ^{13}C NMR (100.62 MHz, D_2O) δ 165.1 (e), 74.2 (t, J = 134.0 Hz, a), 54.1 (h), 42.9 (i), 40.7 (d), 38.7 (k), 31.3 (c), 30.7 (f), 22.8 (t, J = 5.5 Hz, b), 18.9, 18.9 (g, j) ppm; FTIR (KBr) 3423, 3125, 2965, 2885, 2804, 2580, 2360, 1680, 1648, 1588, 1399, 1310, 1146, 1068, 926, 877 cm^{-1}. Anal. calcd for $C_{11}H_{25}N_3O_7P_2.2H_2O$: C, 35.39; H, 6.75; N, 11.26; found: C, 35.58; H, 6.09; N, 11.39.

Preparation of bis(2,3,4,6,7,8-hexahydropyrrolo[1,2-a]pyrimidin-1-ium) (4-amino-1-hydroxybutane-1,1-diyl)bis(hydrogen phosphonate), [DBNH]$_2$[ALN] (**4**)

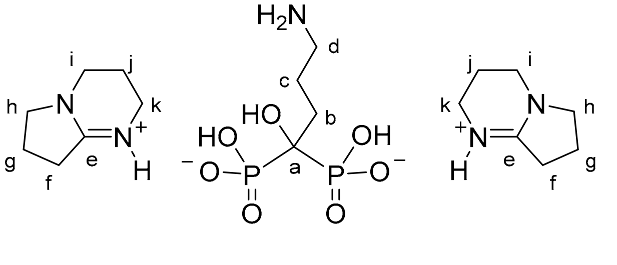

Using 1,5-diazabicyclo(4.3.0)non-5-ene (399 mg, 3.22 mmol) $[DBNH]_2[ALN]$ was obtained as a colorless paste in quantitative yield (800 mg). T_g = 45.7 °C; ^1H NMR (400.13 MHz, D_2O) δ 3.64 (t, J = 7.2 Hz, 4H, k), 3.39 (t, J = 5.7 Hz, 4H, h), 3.34 (t, J = 5.7 Hz, 4H, i), 3.06–2.97 (m, 2H, d), 2.82 (t, J = 8.0 Hz, 4H, f), 2.09 (quint, J = 7.6 Hz, 4H, g), 2.02–1.90 (m, 8H, b, c, j). ^{13}C NMR (100.62 MHz, D_2O) δ 165.0 (e), 74.2 (t, J = 127.4 Hz, a), 54.0 (h), 42.8 (i), 40.7 (d), 38.6 (k), 31.6 (c), 30.6 (f), 23.1 (t, J = 7.0 Hz, b), 18.8, 18.8 (g, j) ppm; FTIR (KBr) 3425, 3224, 3127, 2966, 2887, 2785, 2652, 2555, 2360, 2342, 1681, 1648, 1590, 1401, 1309, 1166, 1069, 979 cm^{-1}. Anal. calcd for $C_{18}H_{37}N_5O_7P_2.5H_2O$: C, 36.80; H, 8.06; N, 11.92; found: C, 36.88; H, 7.97; N, 12.01.

Preparation of (1-(2-hydroxyethyl)-3-methyl-1*H*-imidazol-3-ium) hydrogen (4-amino-1-hydroxy-1-phosphonobutyl)phosphonate, $[C_2OHMIM][ALN]$ (**5**)

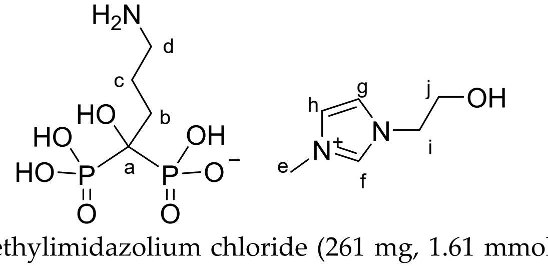

Using hydroxyethylmethylimidazolium chloride (261 mg, 1.61 mmol) $[C_2OHMIM][ALN]$ was obtained as a colorless paste in quantitative yield (660 mg). T_g = 64.5 °C; ^1H NMR (400.13 MHz, D_2O) δ 8.73 (br s, 1H, f), 7.49 (br s, 1H, h), 7.43 (br s, 1H, g), 4.29 (t, J = 4.9 Hz, 2H, i), 3.98–3.84 (m, 5H, e, j), 3.09–2.97 (m, 2H, d), 2.08–1.91 (m, 4H, b, c) ppm. ^{13}C NMR (100.62 MHz, D_2O) δ 124.2 (h), 123.1 (g), 74.1 (t, J = 134.5 Hz, a), 60.4 (j), 52.1 (i), 40.6 (d), 36.3 (e), 31.2 (c), 22.8 (t, J = 6.7 Hz, b) ppm; FTIR (KBr) 3418, 3156, 3112, 2960, 2785, 2552, 2359, 2341, 1640, 1575, 1339, 1167, 1066, 917 cm^{-1}. Anal. calcd for $C_{10}H_{23}N_3O_8P_2.2H_2O$: C, 29.20; H, 6.62; N, 10.22; found: C, 29.13; H, 6.71; N, 10.01.

Preparation of bis(1-(2-hydroxyethyl)-3-methyl-1*H*-imidazol-3-ium) (4-amino-1-hydroxybutane-1,1-diyl)bis(hydrogen phosphonate), $[C_2OHMIM]_2[ALN]$

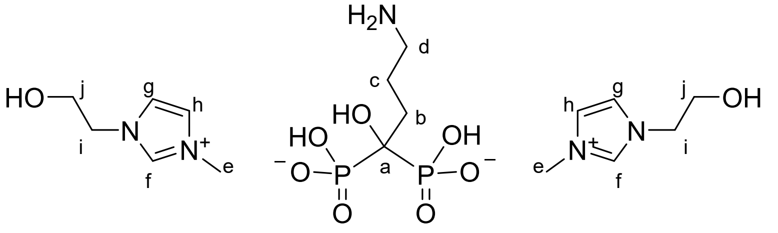

Using hydroxyethylmethylimidazolium chloride (522 mg, 3.22 mmol) $[C_2OHMIM]_2[ALN]$ was obtained as a white solid in quantitative yield (921 mg). T_m = 153.0 °C, T_g = 46.3 °C; ^1H NMR (400.13 MHz, D_2O) δ 7.49 (br s, 2H, h), 7.43 (br s, 2H, g), 4.30 (t, J = 4.9 Hz, 4H, i), 3.96–3.84 (m, 10H, e, j), 3.09–2.97 (m, 2H, d), 2.05–1.87 (m, 4H, b, c) ppm. ^{13}C NMR (100.62 MHz, D_2O) δ 124.2 (h), 123.0 (g), 74.2 (t, J = 127.1 Hz, a), 60.3 (j), 52.1 (i), 40.7 (d), 36.3 (e), 31.6 (c), 23.1 (t, J = 7.1 Hz, b) ppm; FTIR (KBr) 3388, 3149, 3107, 2961, 2881, 2658, 2543, 2360, 2340, 2128, 1645, 1575, 1451, 1398, 1340, 1169, 1072, 976 cm^{-1}. Anal. calcd for $C_{16}H_{33}N_5O_9P_2.H_2O$: C, 37.00; H, 6.79; N, 13.48; found: C, 36.65; H, 7.09; N, 12.87.

Preparation of 2-hydroxy-*N*,*N*,*N*-trimethylethan-1-aminium hydrogen (4-amino-1-hydroxy-1-phosphonobutyl)phosphonate, [Ch][ALN] (**7**)

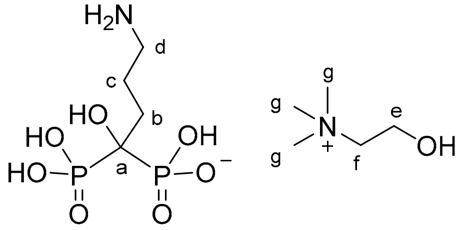

Using choline chloride (224 mg, 1.61 mmol) [Ch][ALN] was obtained as a white solid in quantitative yield (623 mg). T_m = 141.2 °C; T_g = 74.9 °C; ^1H NMR (400.13 MHz, D_2O) δ 4.08–4.00 (m, 2H, e), 3.53–3.46 (m, 2H, f), 3.18 (s, 9H, g), 3.06–2.98 (m, 2H, d), 2.05–1.93 (m, 4H, b, c). ^{13}C NMR (100.62 MHz, D_2O) δ 74.1 (t, J = 133.1 Hz, a), 68.0 (t, J = 3.1 Hz, f), 56.2 (e), 54.5 (t, J = 4.0 Hz, g), 40.6 (d), 31.2 (c), 22.8 (t, J = 6.8 Hz, b) ppm; FTIR (KBr) 3423, 3253, 3019, 2967, 2923, 2857, 2785, 2550, 2359, 2341,

2101, 1646, 1489, 1478, 1400, 1163, 1059, 956, 919 cm^{-1}. Anal. calcd for $C_9H_{26}N_2O_8P_2.2H_2O$: C, 27.84; H, 7.79; N, 7.21; found: C, 27.43; H, 7.74; N, 6.91.

Preparation of bis(2-hydroxy-*N*,*N*,*N*-trimethylethan-1-aminium) (4-amino-1-hydroxybutane-1,1-diyl)bis(hydrogen phosphonate), $[Ch]_2[ALN]$ (**8**)

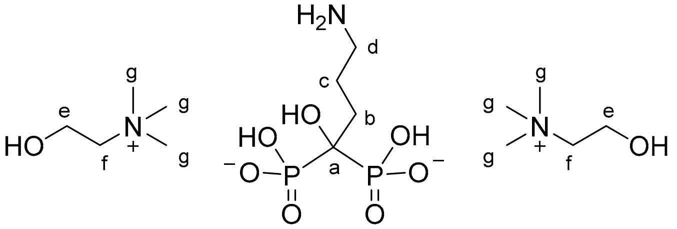

Using choline chloride (447 mg, 3.22 mmol) $[Ch]_2[ALN]$ was obtained as a colorless paste in quantitative yield (848 mg). T_g = 63.8 °C; ^1H NMR (400.13 MHz, D_2O) δ 4.10–4.02 (m, 4H, e), 3.55–3.48 (m, 4H, f), 3.19 (s, 18H, g), 3.08 – 3.00 (m, 2H, d), 2.03–1.93 (m, 4H, b, c). ^{13}C NMR (100.62 MHz, D_2O) δ 74.2 (t, J = 127.0 Hz, a), 68.1 (t, J = 3.8 Hz, f), 56.2 (e), 54.5 (t, J = 4.0 Hz, g), 40.7 (d), 31.6 (c), 23.1 (t, J = 7.0 Hz, b) ppm; FTIR (KBr) 3266, 3018, 2959, 2901, 2785, 2663, 2528, 2357, 2342, 2137, 1646, 1478, 1346, 1267, 1167, 1090, 1057, 1006, 970, 949 cm^{-1}. Anal. calcd for $C_{14}H_{39}N_3O_9P_2.7H_2O$: C, 28.92; H, 9.19; N, 7.23; found: C, 28.48; H, 9.02; N, 6.90.

NMR Spectra of ALN-OSILs

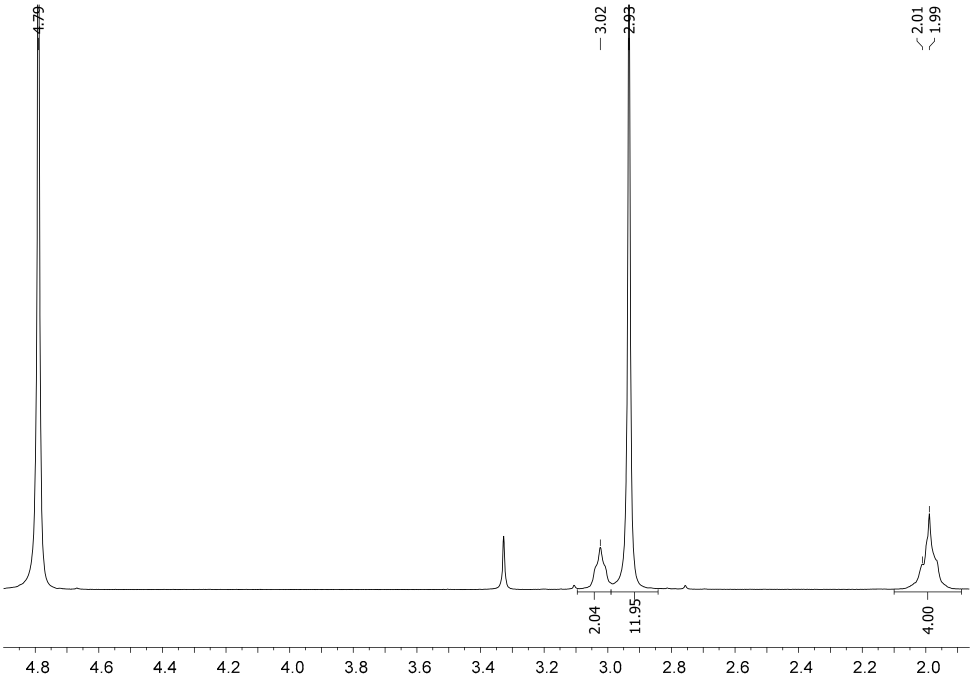

Figure S1. ^1H NMR spectra of [TMGH][ALN].

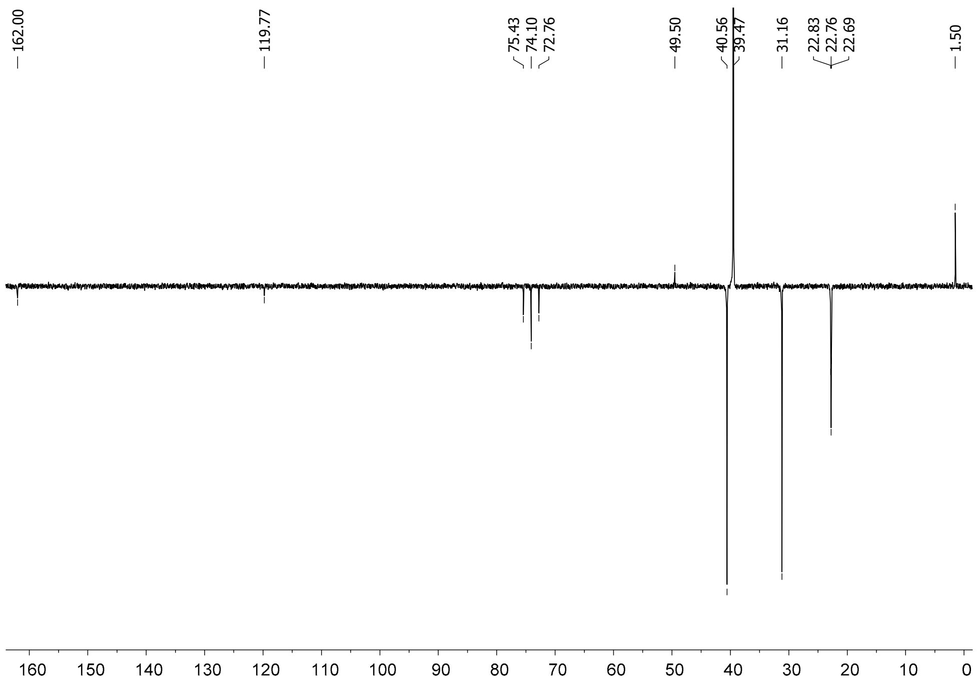

Figure S2. ^{13}C NMR spectra of [TMGH][ALN].

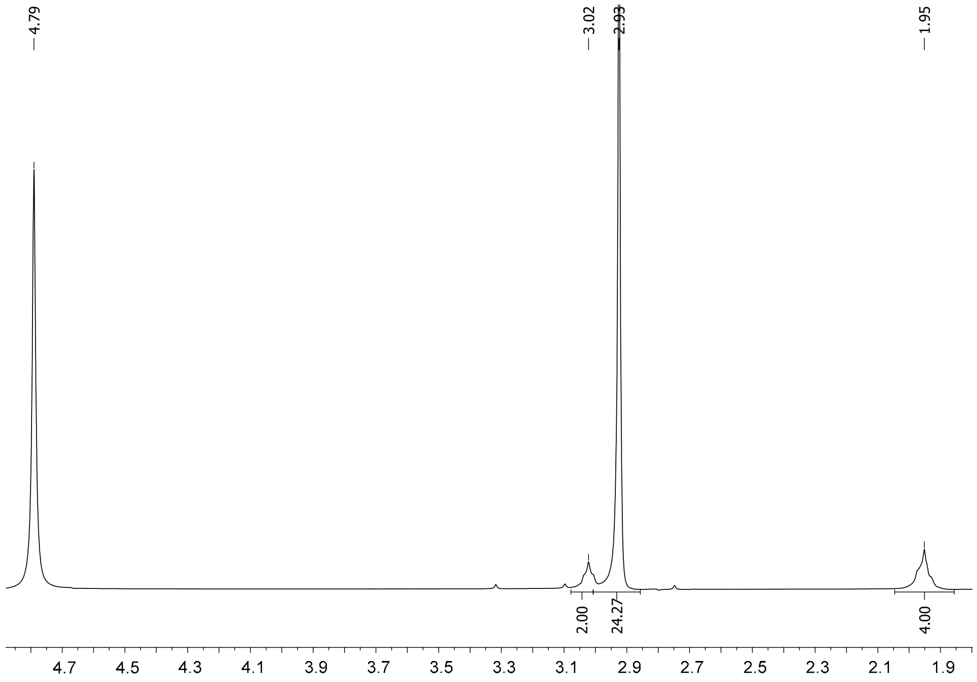

Figure S3. ^{1}H NMR spectra of $[TMGH]_2[ALN]$.

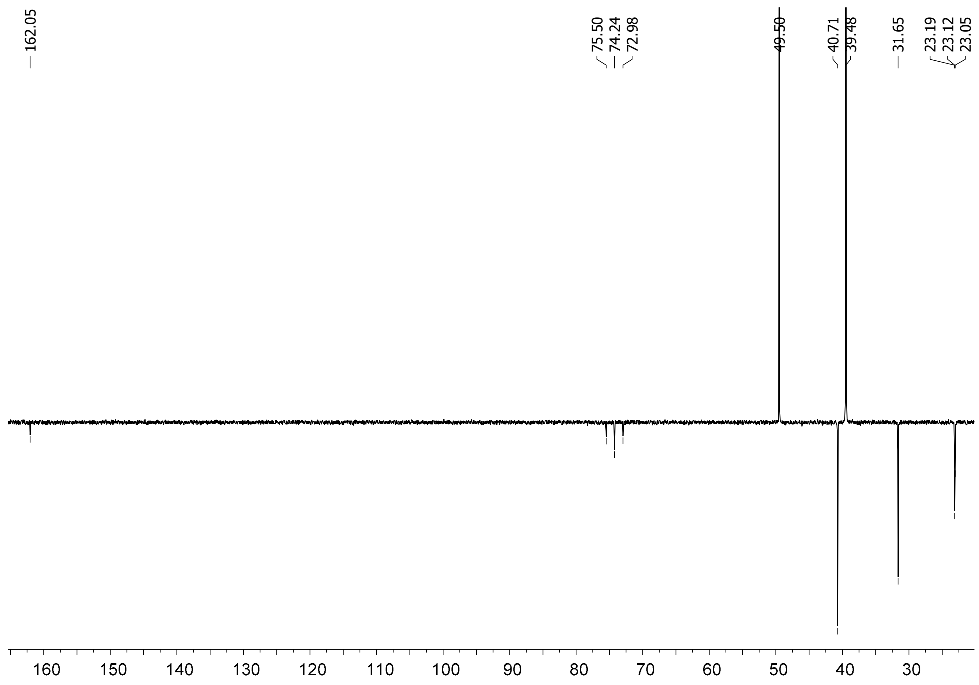

Figure S4. ^{13}C NMR spectra of $[TMGH]_2[ALN]$.

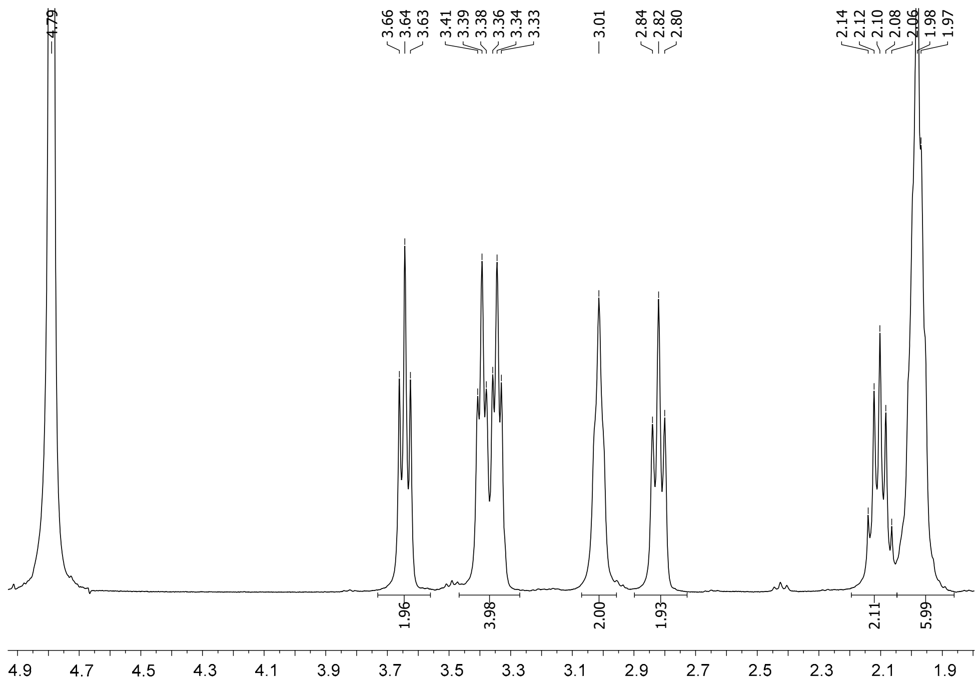

Figure S5. ^{1}H NMR spectra of [DBNH][ALN].

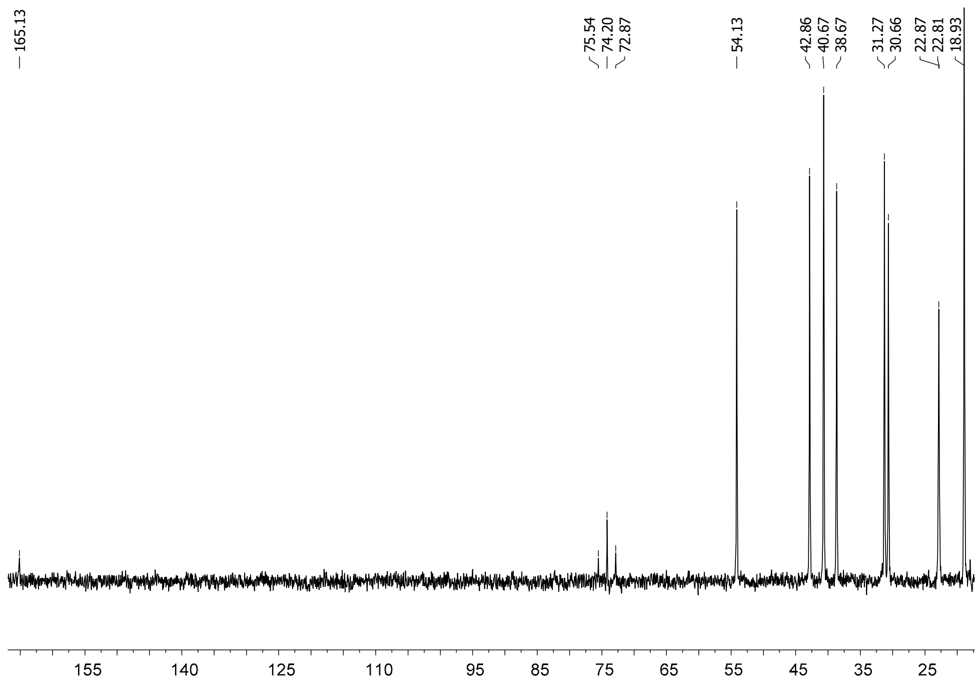

Figure S6. ^{13}C NMR spectra of [DBNH][ALN].

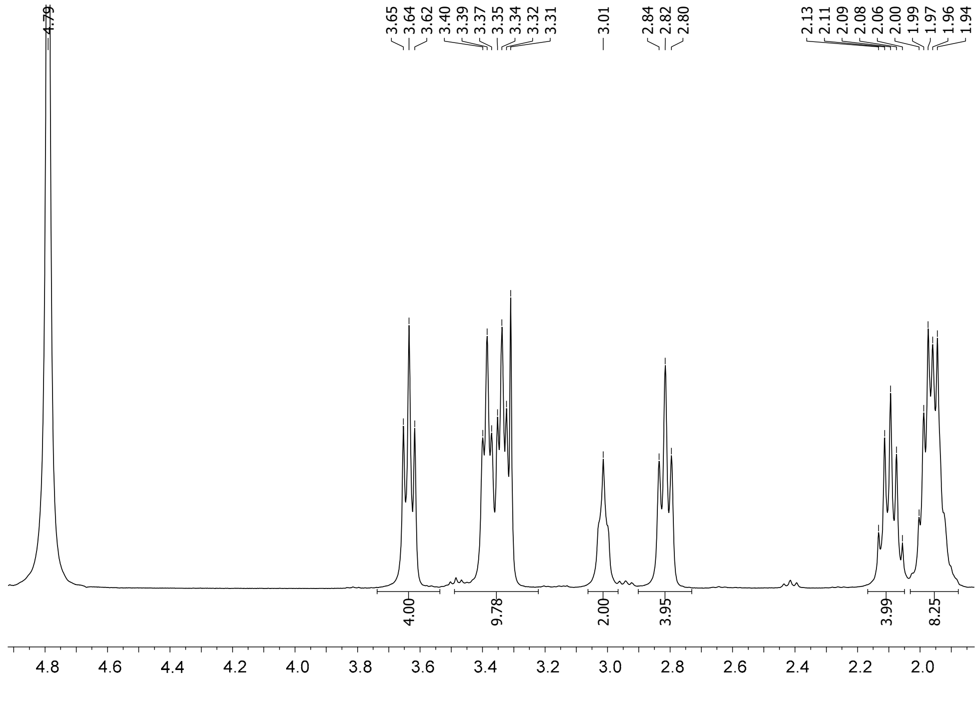

Figure S7. ^{1}H NMR spectra of $[DBNH]_2[ALN]$.

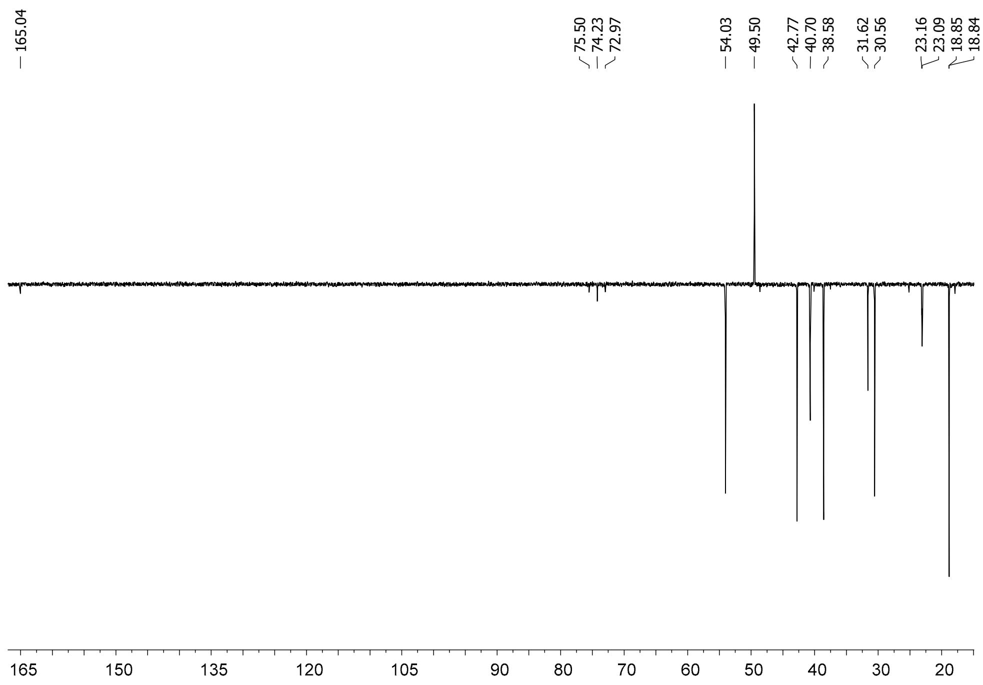

Figure S8. ^{13}C NMR spectra of $[DBNH]_2[ALN]$.

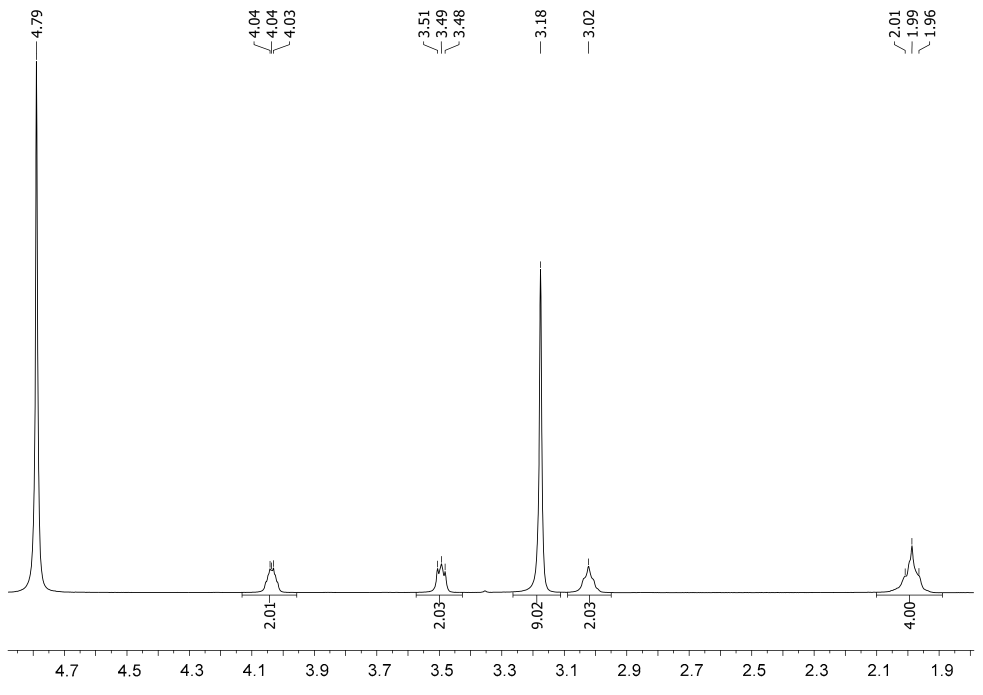

Figure S9. ^{1}H NMR spectra of [Ch][ALN].

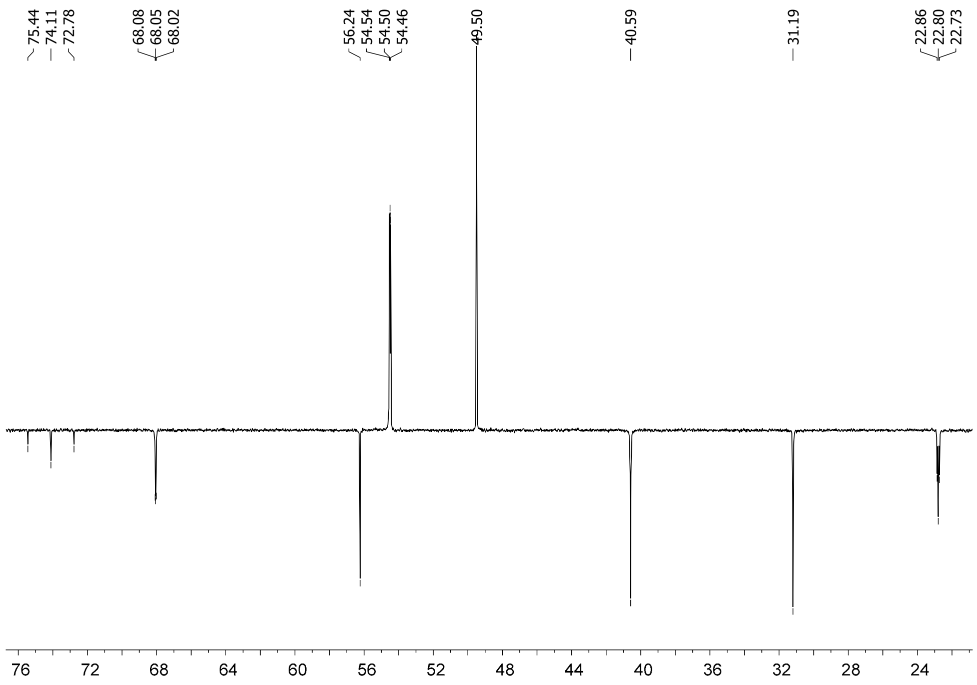

Figure S10. ^{13}C NMR spectra of [Ch][ALN].

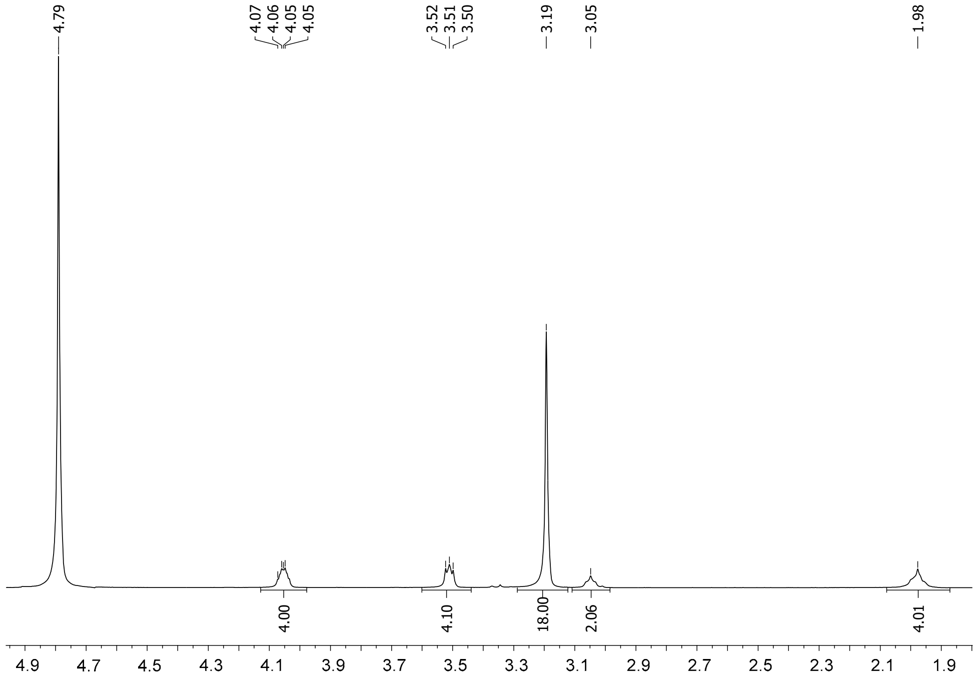

Figure S11. ^{1}H NMR spectra of $[Ch]_2[ALN]$.

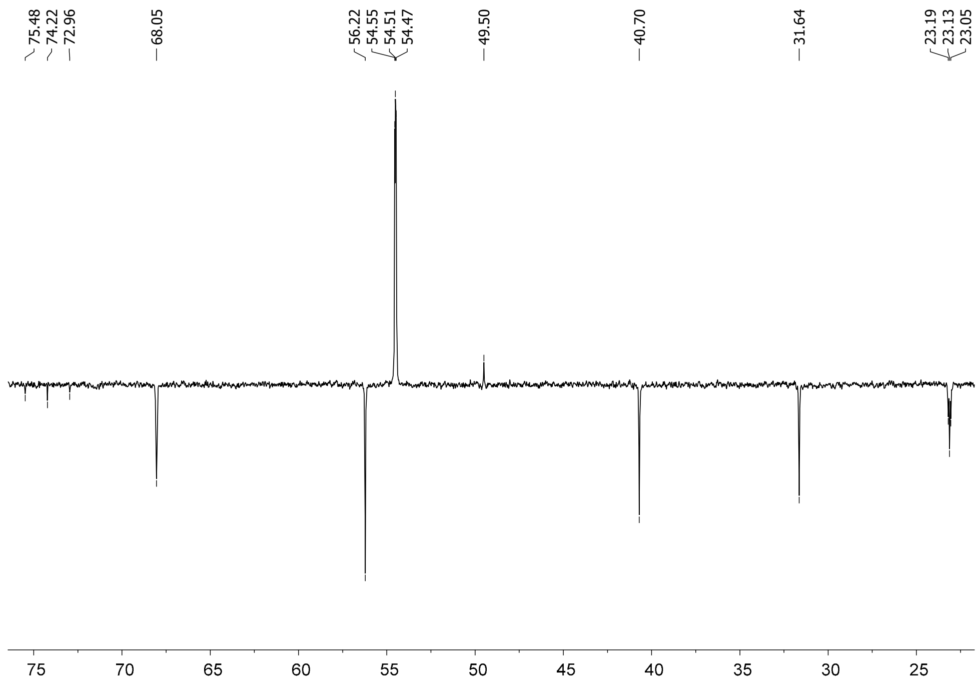

Figure S12. ^{13}C NMR spectra of $[Ch]_2[ALN]$.

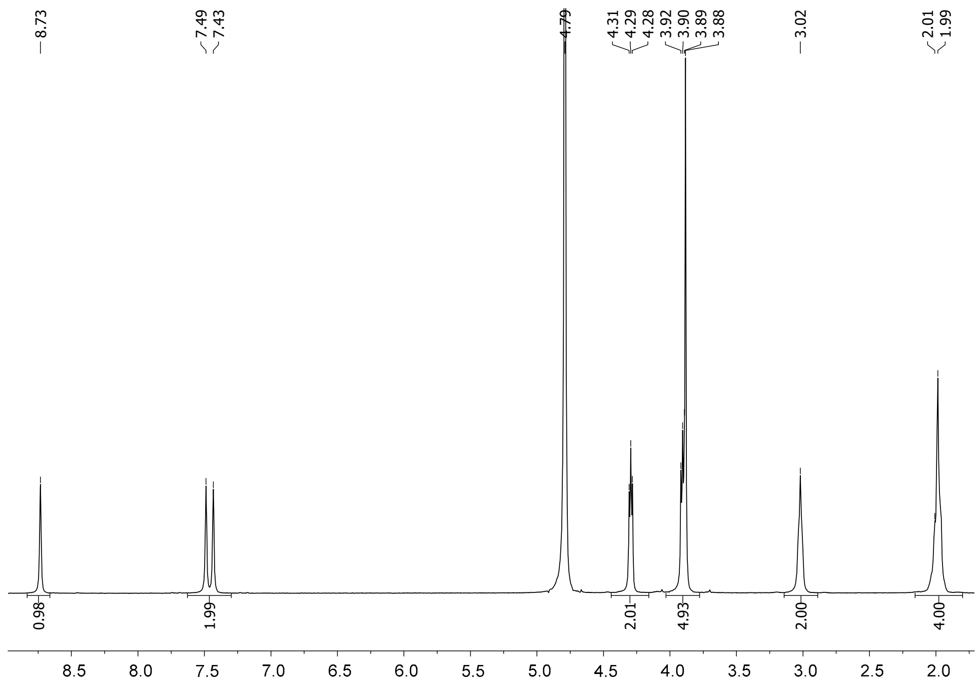

Figure S13. ^{1}H NMR spectra of $[C_2OHMIM][ALN]$.

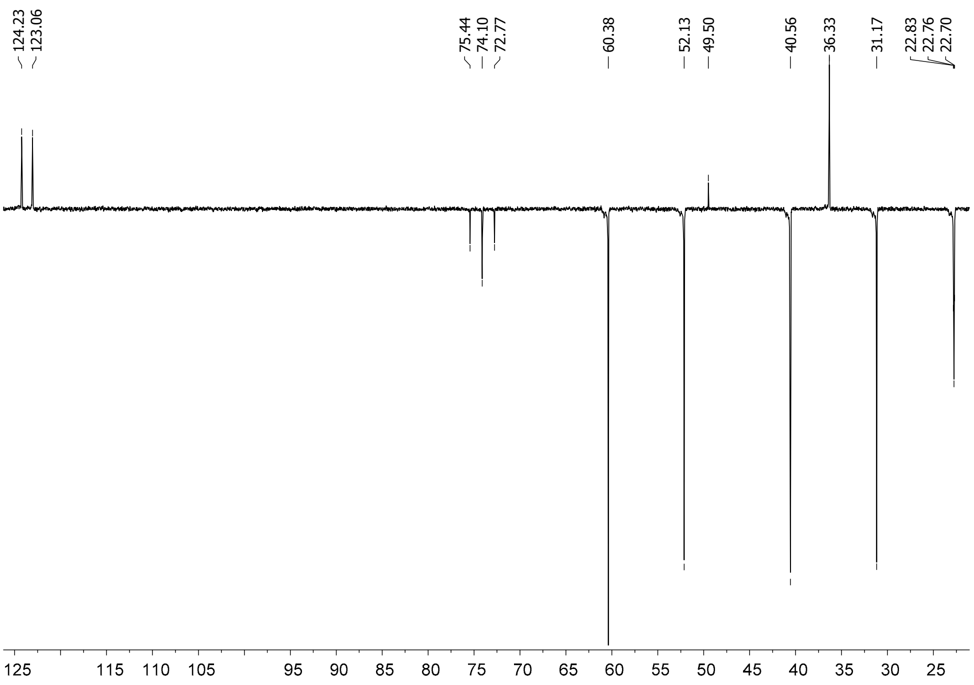

Figure S14. ^{13}C NMR spectra of [C_2OHMIM][ALN].

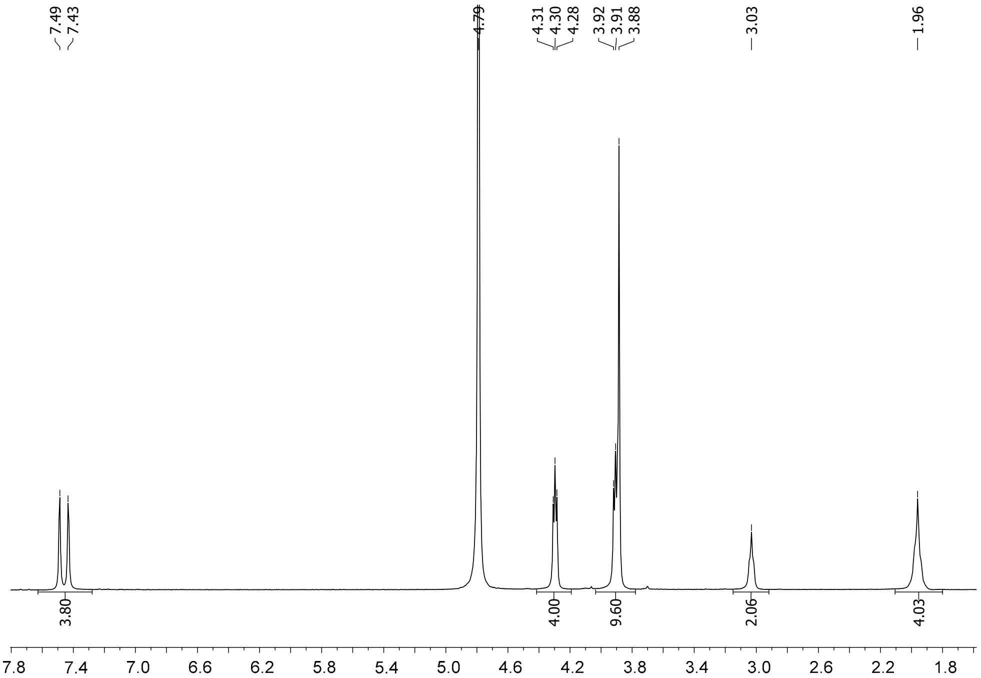

Figure S15. ^{1}H NMR spectra of [C_2OHMIM]$_2$[ALN].

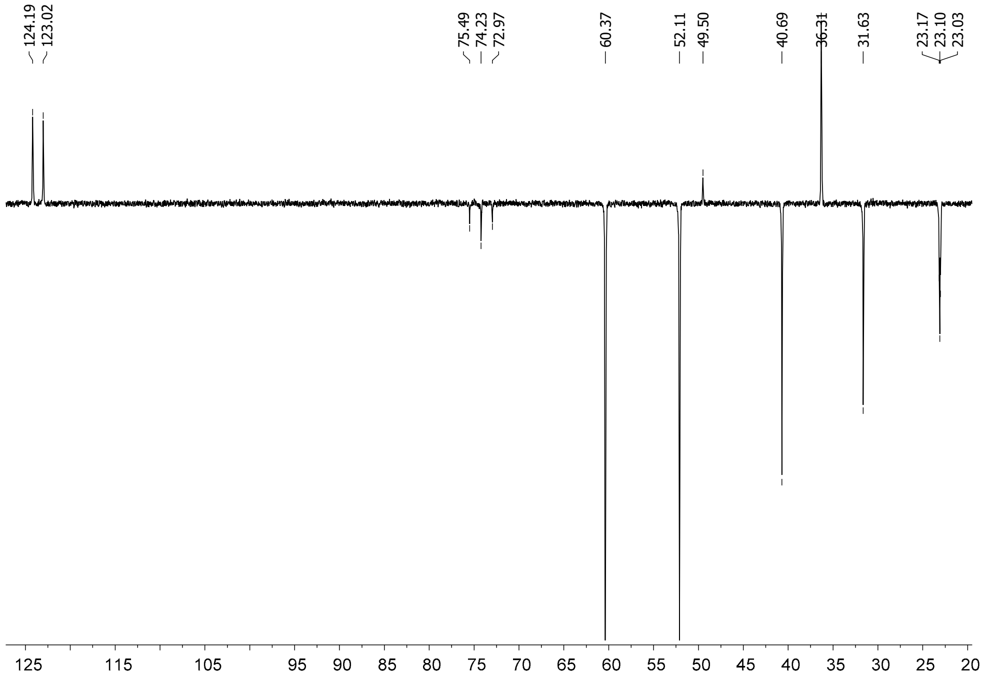

Figure S16. ^{13}C NMR spectra of [C_2OHMIM]$_2$[ALN].

FTIR Spectra of ALN-OSILs

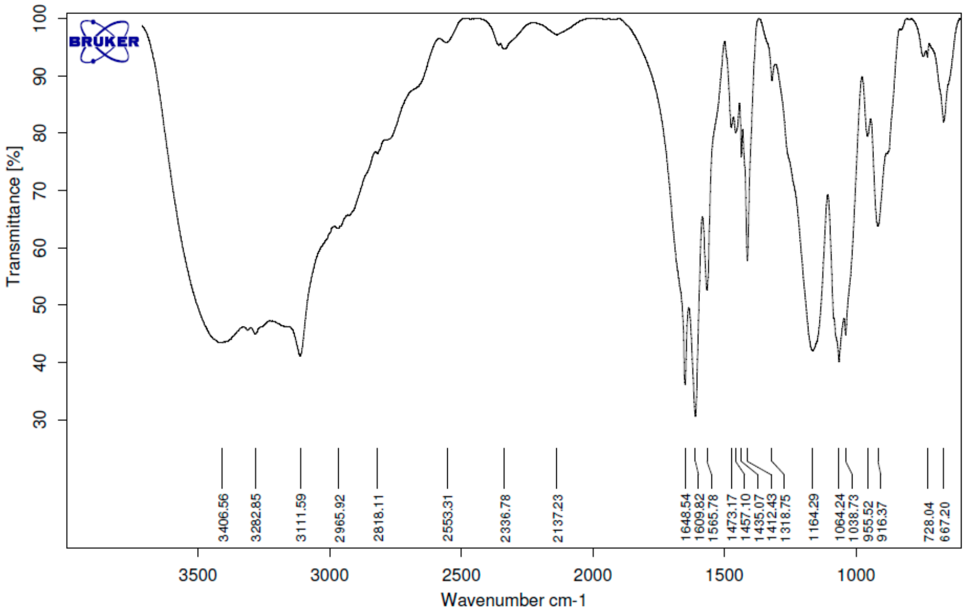

Figure S17. FTIR spectra of [TMGH][ALN].

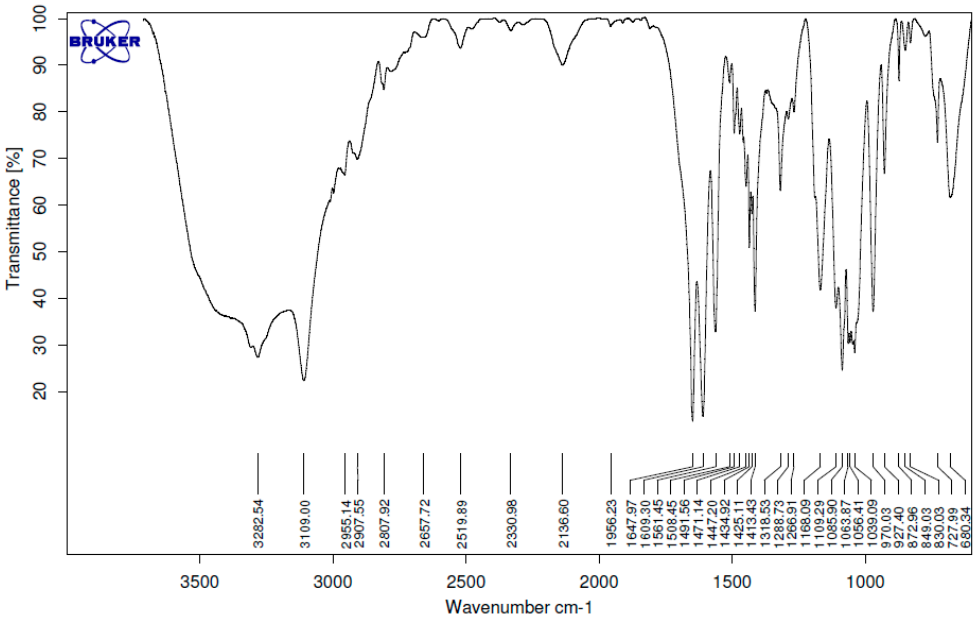

Figure S18. FTIR spectra of [TMGH]$_2$[ALN].

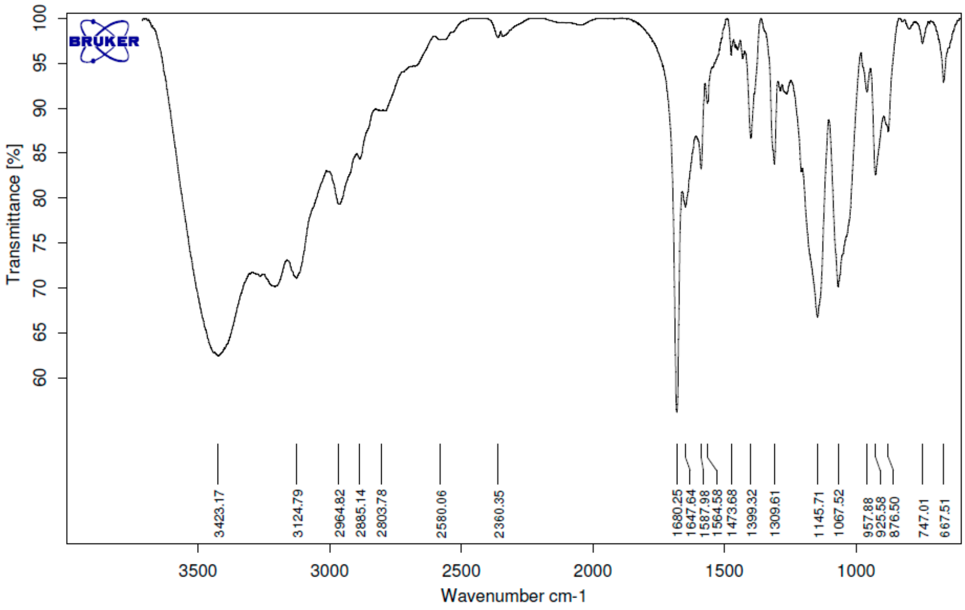

Figure S19. FTIR spectra of [DBNH][ALN].

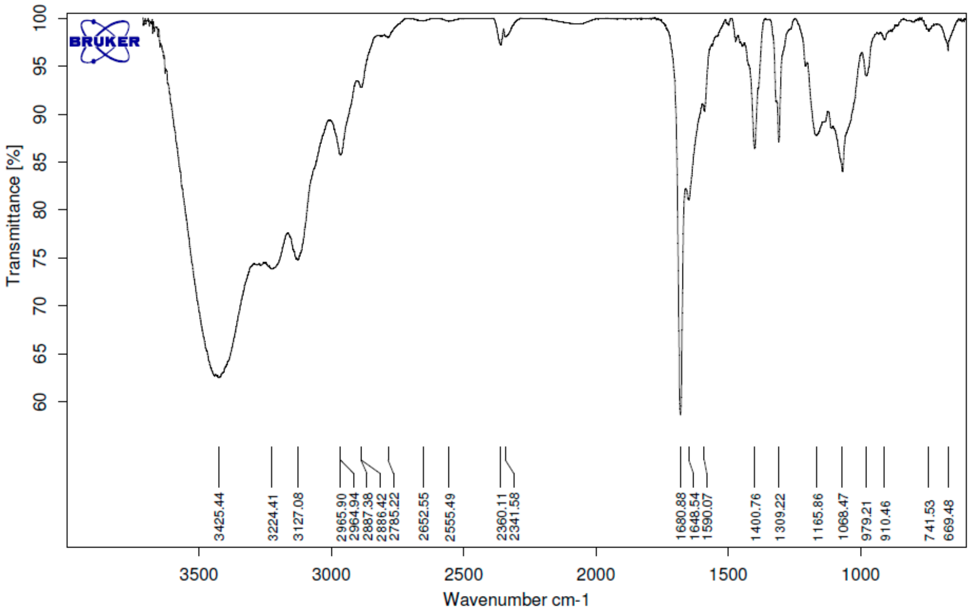

Figure S20. FTIR spectra of $[DBNH]_2[ALN]$.

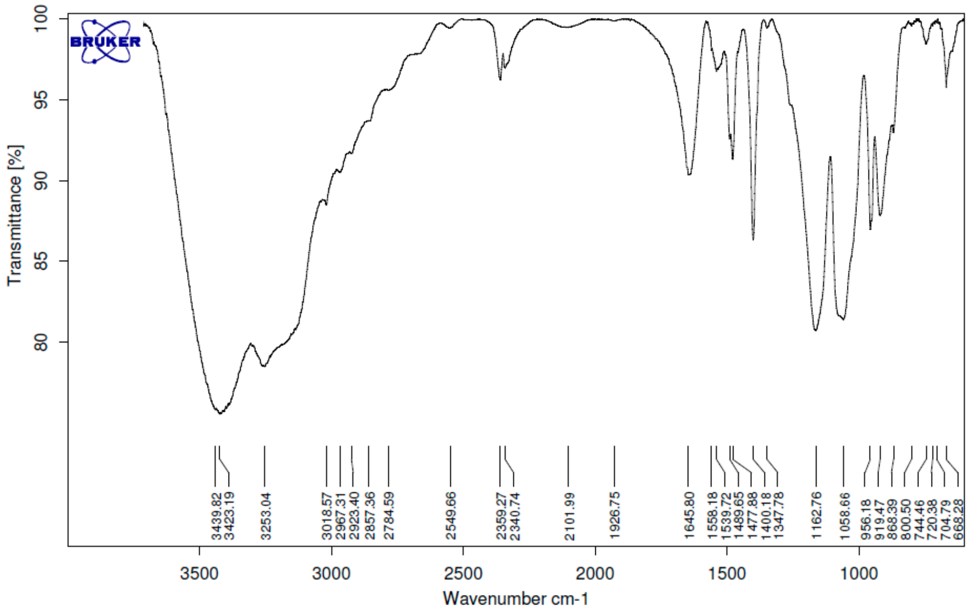

Figure S21. FTIR spectra of [Ch][ALN].

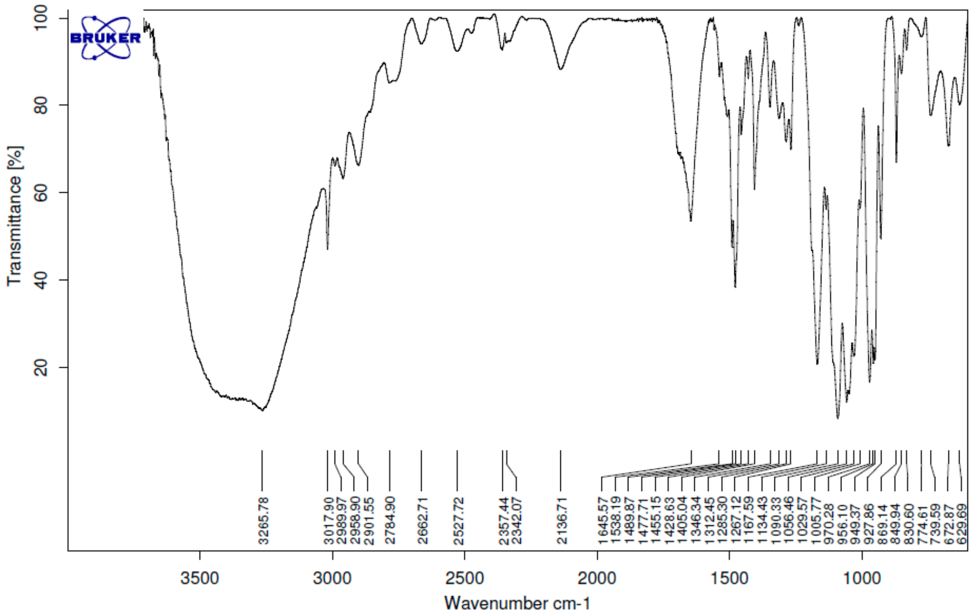

Figure S22. FTIR spectra of $[Ch]_2[ALN]$.

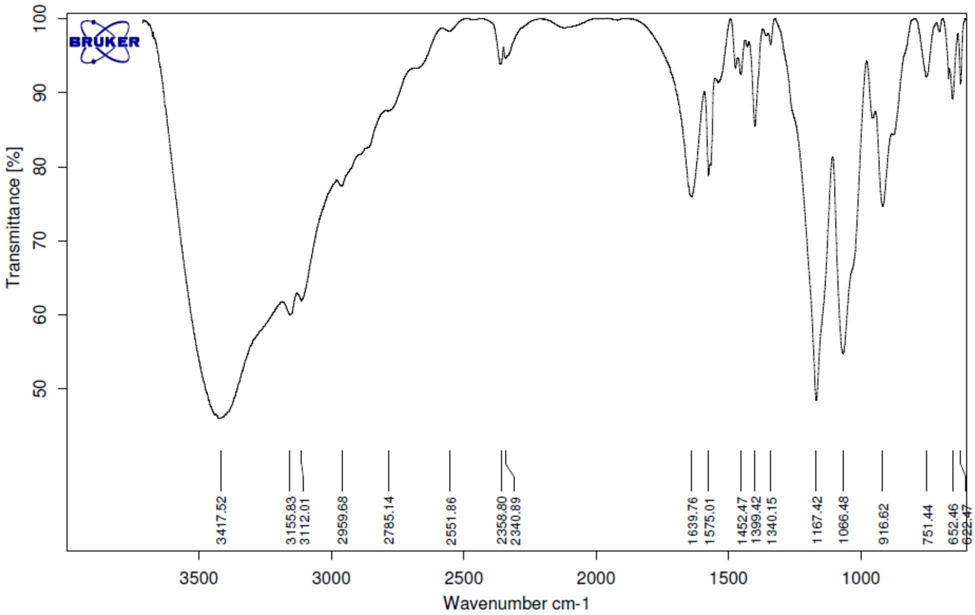

Figure S23. FTIR spectra of [C2OHMIM][ALN].

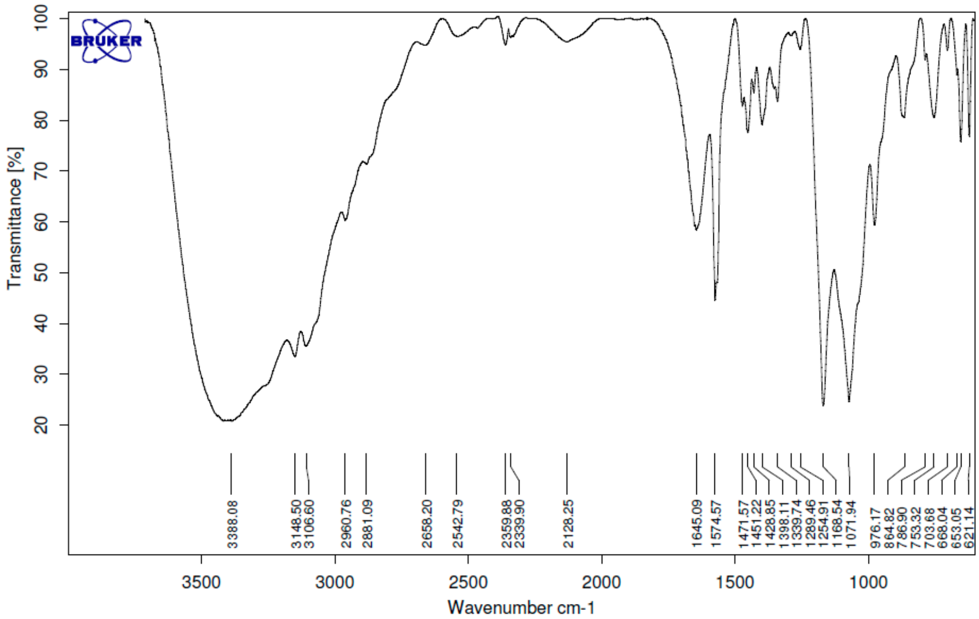

Figure S24. FTIR spectra of [C2OHMIM]2[ALN].

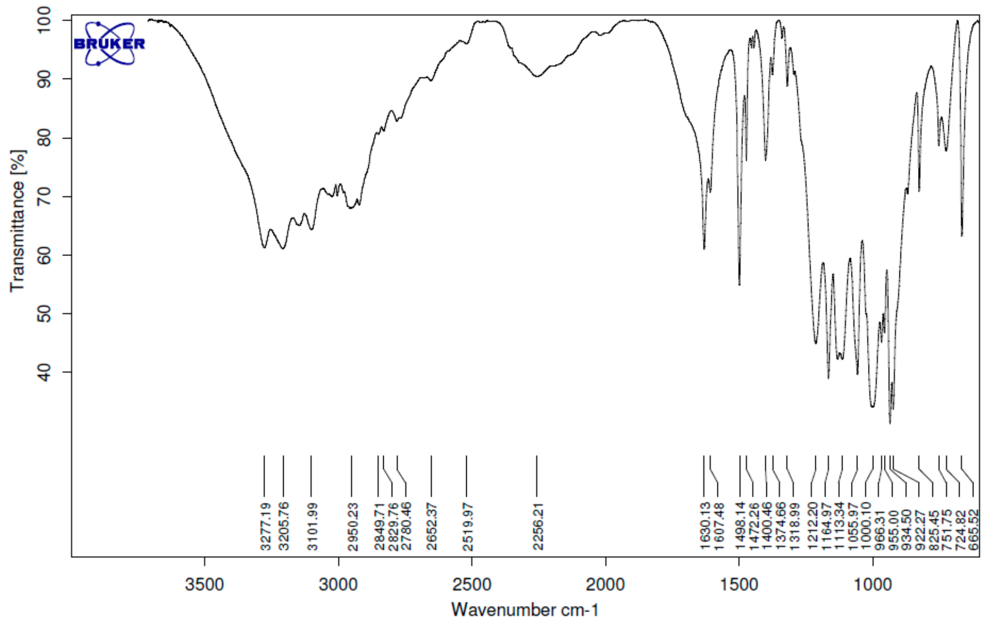

Figure S25. FTIR spectra of alendronic acid.

DSC Thermograms of ALN-OSILs

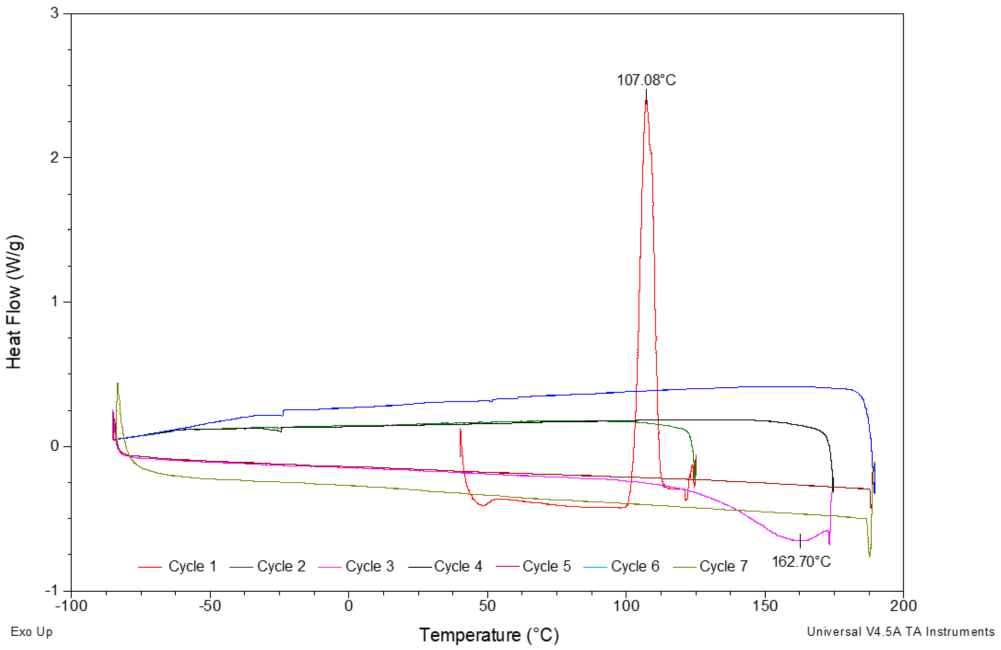

Figure S26. DSC thermogram of [TMGH][ALN].

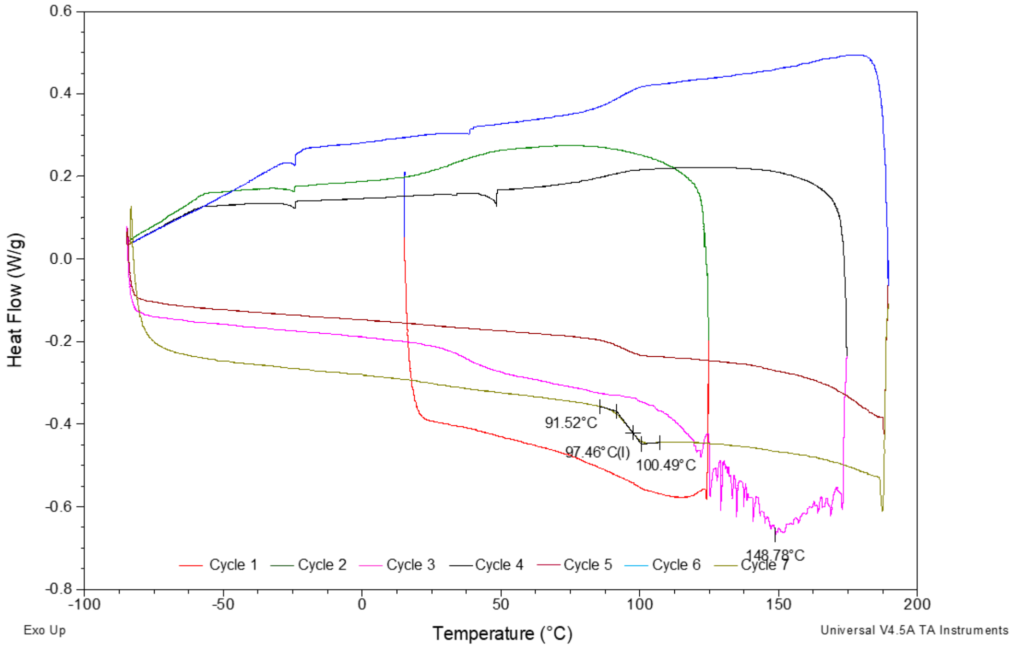

Figure S27. DSC thermogram of $[TMGH]_2[ALN]$.

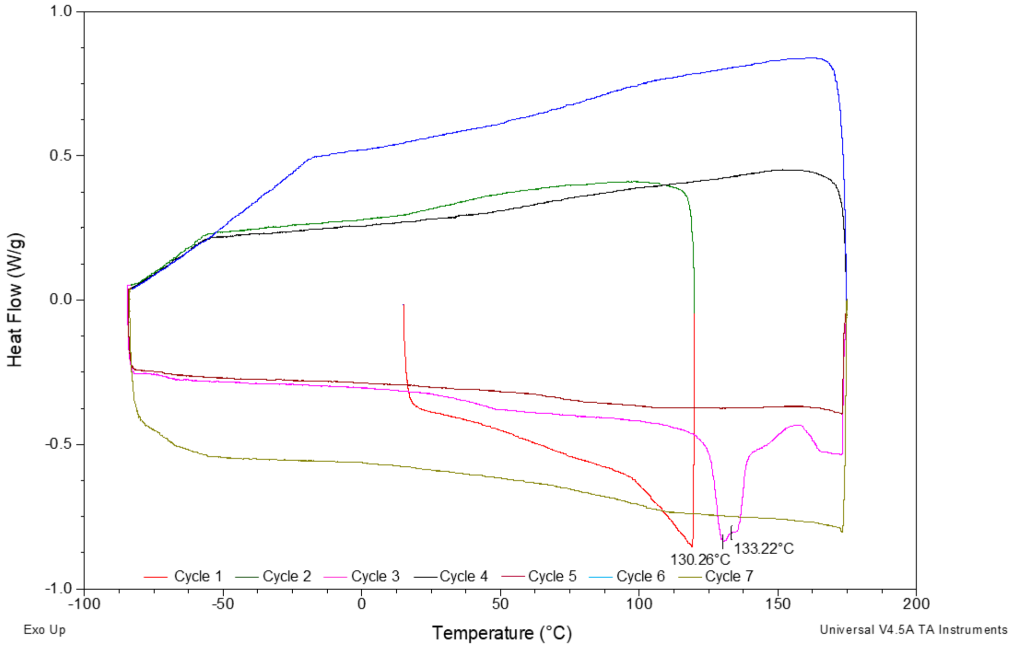

Figure S28. DSC thermogram of [DBNH][ALN].

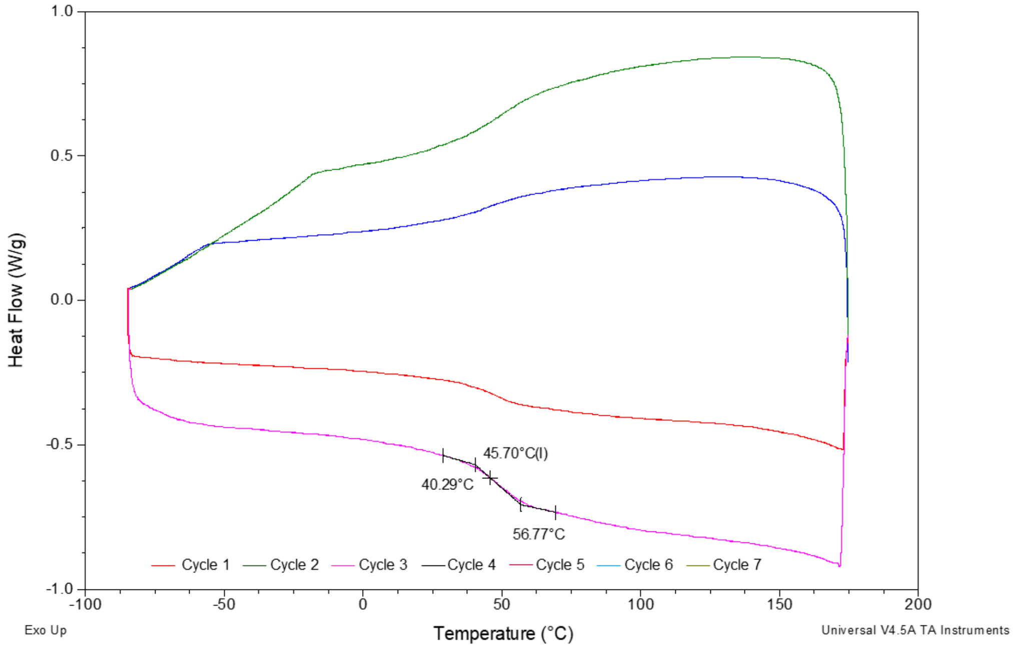

Figure S29. DSC thermogram of $[DBNH]_2[ALN]$.

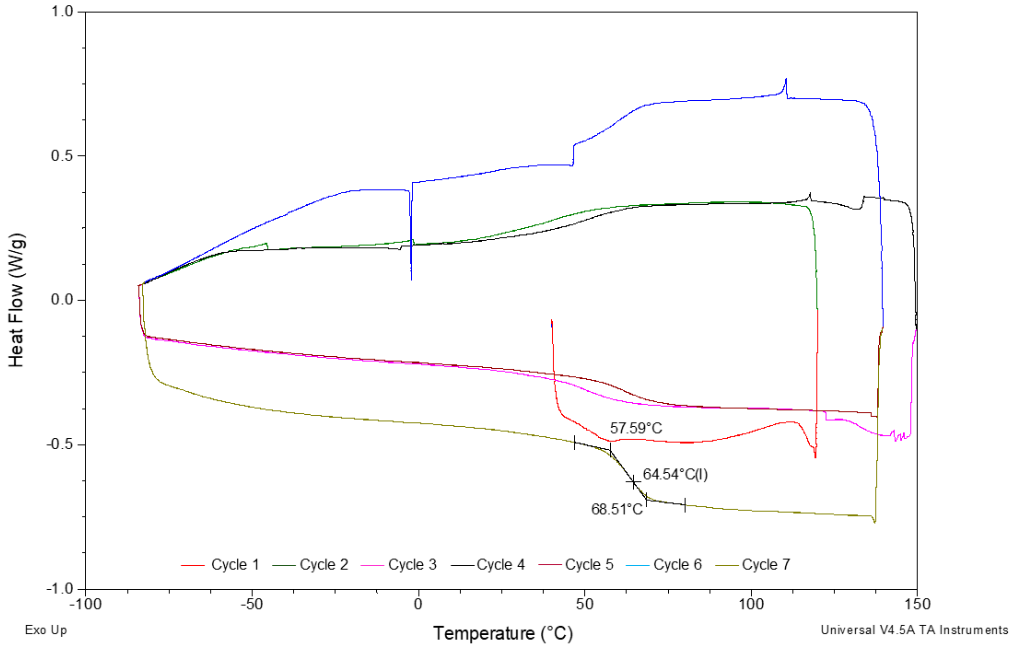

Figure S30. DSC thermogram of $[C_2OHMIM][ALN]$.

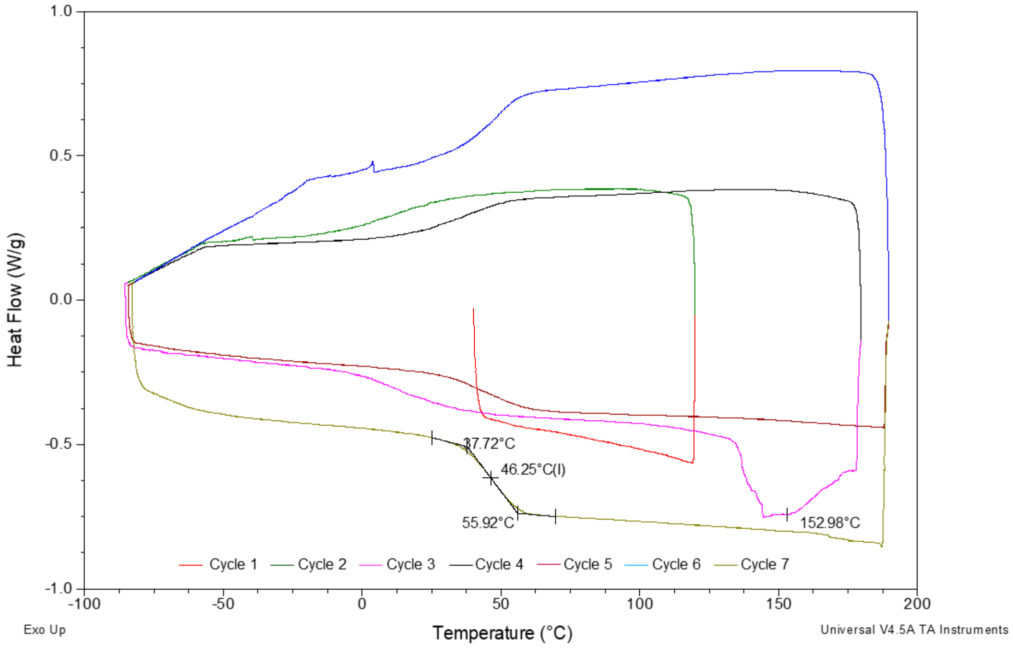

Figure S31. DSC thermogram of $[C_2OHMIM]_2[ALN]$.

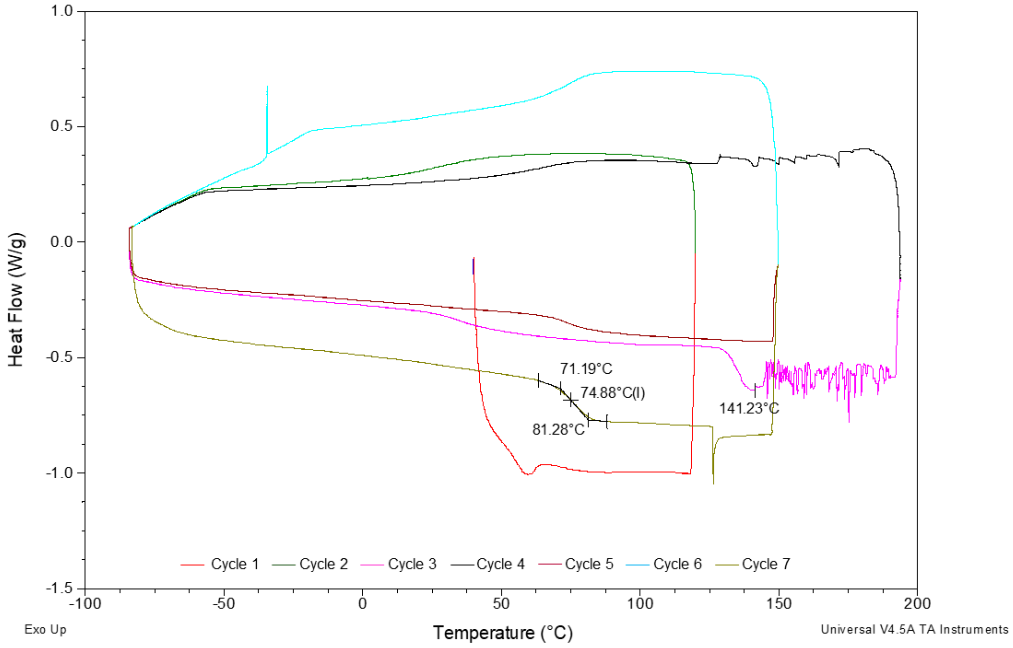

Figure S32. DSC thermogram of [Ch][ALN].

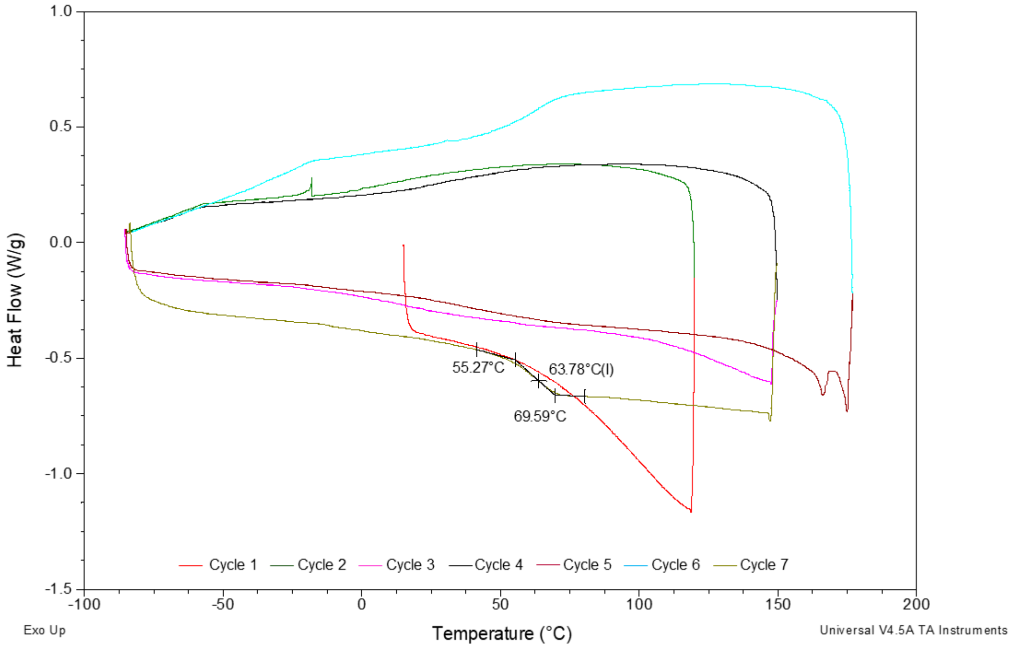

Figure S33. DSC thermogram of $[Ch]_2[ALN]$.

Cytotoxicity Studies

The antiproliferative effects of the developed OSILs was assessed on different cell types: human gingival fibroblasts (non-neoplastic control), ductal breast epithelial cancer cells (T47D cell line), lung carcinoma cells (A549 cell line) and osteosarcoma cells (MG63 cell line). Cells were seeded at 10^4 cells/cm^2, and maintained in α-minimal essential medium (a-MEM) supplemented with 10% fetal bovine serum, 100 IU/mL penicillin, 2.5 μg/mL streptomycin, 2.5 μg/mL amphotericin B, and 50 μg/mL ascorbic acid. After 24 h of incubation, culture medium was renewed and supplemented with the different ALN-containing OSILs. Paclitaxel, a well-known cytotoxic agent, was used as a positive control. Cell cultures were maintained in a 5% CO_2 humidified atmosphere at 37 °C for 24 h and 72 h.

Cellular viability/proliferation was evaluated by MTT assay, which relies in the reduction of 3-(4,5-dimethylthiazol-2-yl)-2,5-diphenyltetrazolium bromide to a purple formazan product by viable cells. Half-maximal inhibitory concentration (IC_{50}) values were calculated by means of a nonlinear regression analysis of concentration-effect curves, using GraphPad Prism software (version 2012).
